# Decoding pyroptosis at single-cell resolution: heterogeneity, microenvironment remodeling and potential translational insights

**DOI:** 10.3389/fimmu.2026.1869338

**Published:** 2026-10-22

**Authors:** Lingyan Xiang, Na Tang, Yaqi Deng, Jingping Yuan, Jie Rao, Aolong Ma, Qianwen Wang, Li Xu, Lina Zhao, Honglin Yan

**Affiliations:** 1 Department of Pathology, Renmin Hospital of Wuhan University, Wuhan, Hubei, China; 2 Department of Pathology, Maternal and Child Health Hospital of Hubei Province, Tongji Medical College, Huazhong University of Science and Technology, Wuhan, Hubei, China

**Keywords:** heterogeneity, microenvironment remodeling, prognosis, pyroptosis, single-cell RNA sequencing, therapy

## Abstract

Pyroptosis, a pro-inflammatory programmed cell death mediated by inflammatory caspases and Gasdermin (GSDM) family proteins, exerts dual roles in host defense and pathological damage. Traditional bulk RNA sequencing (bulk RNA-seq) is limited in capturing single-cell heterogeneity and dissecting intercellular communication, impeding in-depth mechanistic exploration of pyroptosis. Single-cell RNA sequencing (scRNA-seq) has emerged as a powerful tool to address these limitations, enabling high-throughput profiling of single-cell transcriptomes and precise characterization of gene expression heterogeneity, cell subset features, and putative intercellular interaction networks. Here, we comprehensively summarize the cutting-edge applications of scRNA-seq in pyroptosis research from four core aspects: (1) dissecting pyroptosis-related transcriptional heterogeneity with cell subtype and state specificity across diverse cell types and diseases; (2) exploring candidate mechanisms underlying pyroptosis-associated microenvironment remodeling (including immune polarization and stromal reorganization) in pathological progression; (3) harnessing scRNA-seq to construct prognostic pyroptosis-related gene signatures (PRGS) for candidate risk stratification via integration with bulk RNA-seq data; and (4) putative targeted therapeutic strategies (preclinical-stage evidence), including modulation of core pyroptotic nodes, regulation of endogenous modulators, exploitation of exogenous compounds, and indirect interventions through cell function optimization or gut microbiota modulation. Future research should integrate scRNA-seq with multi-omics technologies to construct a multi-dimensional pyroptosis regulatory network, thereby supporting the preclinical development and potential clinical translation of pyroptosis-targeted therapies for inflammation-associated diseases and tumors.

## 1 Introduction

Pyroptosis is a form of programmed cell death driven by inflammatory caspases, characterized by altered membrane permeability, osmotic lysis, and release of pro-inflammatory cytokines (such as IL-1β and IL-18). Unlike apoptosis, pyroptosis induces strong inflammatory responses through membrane pore formation mediated by the gasdermin (GSDM) protein family, particularly GSDMD (1, 2). This process has been confirmed to be closely associated with infection, autoimmune diseases, and tumor progression (3, 4). Since its naming, significant progress has been made in understanding the molecular mechanisms of pyroptosis: the classical pathway involves activation of caspase-1 by NLRP3 or AIM2 inflammasomes, which then cleaves GSDMD to release inflammatory cytokines (5–7); the non-classical pathway directly activates caspase-4/5/11 via bacterial lipopolysaccharide (LPS), inducing pyroptosis bypassing inflammasomes (8, 9). Notably, pyroptosis exhibits dual functions in host defense and pathological damage: moderate activation enhances host defense, while excessive activation may exacerbate inflammatory injuries in sepsis, neurodegenerative diseases, or promote immune escape by remodeling the tumor microenvironment (10–12).

However, traditional research methods have significant limitations in dissecting pyroptosis mechanisms. Population-level detection techniques (such as western blot and bulk RNA sequencing) fail to capture single-cell heterogeneity. For example, in the tumor microenvironment, the expression of pyroptosis-related genes is highly heterogeneous (13), and traditional methods cannot accurately identify the functional states of specific cell subsets due to signal averaging, potentially obscuring differences in pyroptotic activity between malignant cells and immune cells. Additionally, the paracrine effects of pyroptotic cells may reshape the local immune landscape (14, 15), but traditional methods cannot precisely analyze intercellular communication networks, making it difficult to reveal dynamic gene expression changes and cell-microenvironment interaction mechanisms during pyroptosis.

The innovation of single-cell RNA sequencing (scRNA-seq) technology has provided a revolutionary tool to overcome these limitations. This technology isolates single cells using microfluidic chips or droplet sorting, followed by reverse transcription, cDNA amplification, and high-throughput sequencing to achieve high-throughput detection of single-cell transcriptomes (16, 17). Its core advantages lie in resolving the expression heterogeneity of pyroptosis-related genes, inflammatory cytokines, and signaling pathways at the single-cell resolution, identifying pyroptosis-related subsets with unique gene expression profiles, inferring the continuous dynamic process of pyroptosis via pseudotime analysis, and predicting ligand-receptor interaction networks between pyroptotic cells and adjacent cells (such as immune cells). These advantages break through the limitations of population-level detection, enabling the identification of candidate pyroptosis-related cell subsets, temporal inference of key regulatory nodes, and hypothesis-generating dissection of microenvironmental interactions.

Against this background, the present work summarizes recent advances in this field. It should be noted that this is a narrative review rather than a formal systematic review. Literature was curated from PubMed and Web of Science using keywords including pyroptosis, scRNA-seq, single-cell RNA sequencing, microenvironment. We prioritized peer-reviewed original articles published between January 2022 and December 2025 that focus on single-cell transcriptomic studies of pyroptosis; conference abstracts and non-English articles were excluded. No formal PRISMA checklist was applied for literature retrieval and selection.

This review focuses on the cutting-edge applications of scRNA-seq in pyroptosis research, discussing new regulatory mechanisms of pyroptosis at the single-cell resolution and their translational potential in precision medicine from four dimensions: pyroptotic heterogeneity analysis, microenvironment remodeling, disease prognosis, and treatment associations (**Figure 1**). It aims to provide new research perspectives for developing pyroptosis-targeted interventions in disease therapy.

**Figure 1:**
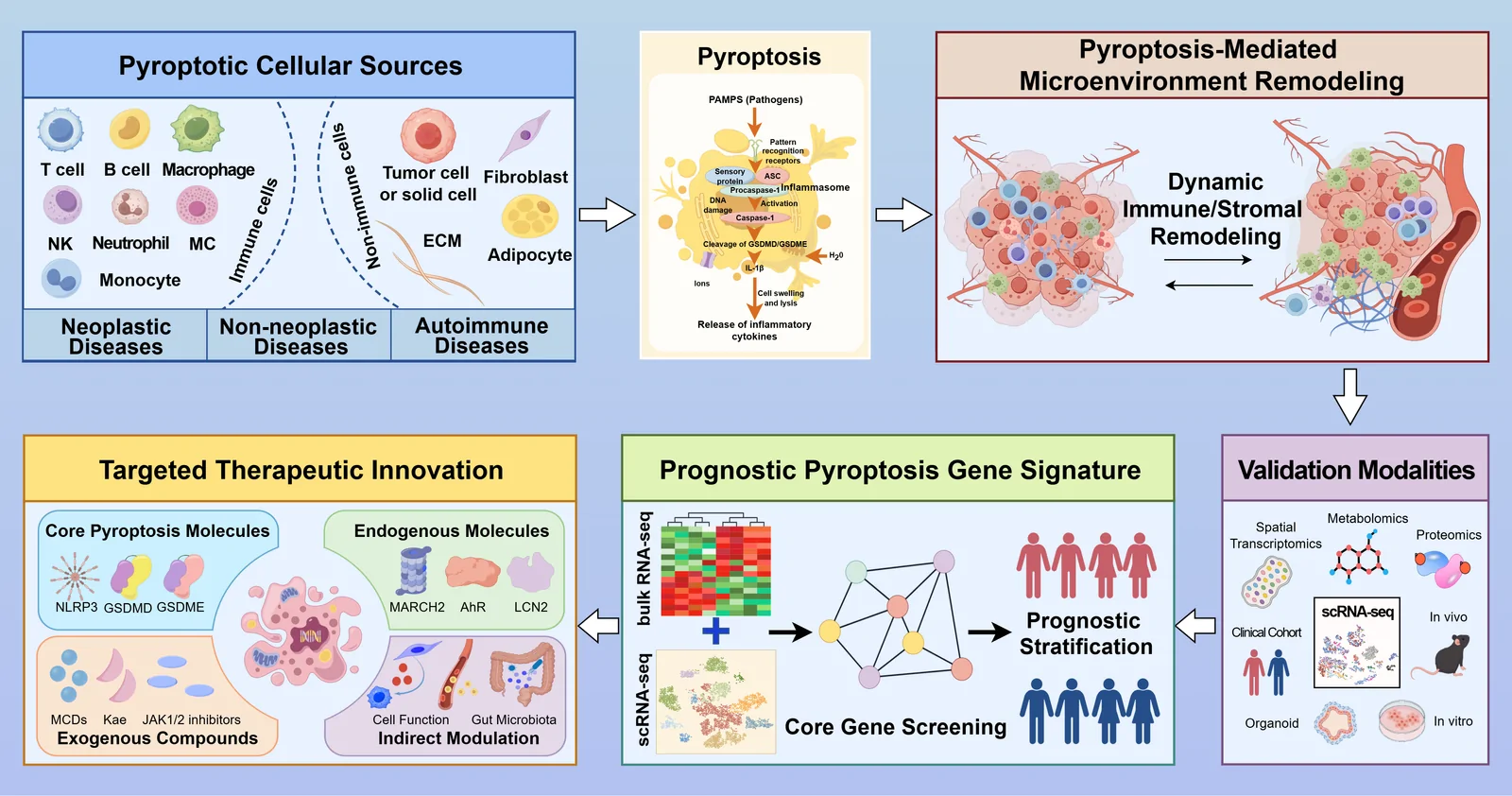
Schematic overview of pyroptosis at single-cell resolution: pyroptotic cellular heterogeneity, microenvironment remodeling and translational insights. This schematic outlines the full regulatory cascade of pyroptosis across disease contexts. Cell-type-specific pyroptotic sources are proposed to activate the inflammasome-caspase-gasdermin execution axis, trigger release of pro-inflammatory cytokines and DAMPs, and drive dynamic immune/stromal microenvironment remodeling and spatial niche reprogramming. With single-cell RNA sequencing as the core, integrated multi-omic strategies (spatial transcriptomics, proteomics, metabolomics) combined with functional assays and clinical cohort validation support the discovery of pyroptosis gene signatures, enabling candidate prognostic stratification and targeted therapeutic development. scRNA-seq, single-cell RNA sequencing; NK, natural killer; MC, mast cell; ECM, extracellular matrix; PAMPs, pathogen-associated molecular patterns.

## 2 Pyroptotic heterogeneity at single-cell resolution

As a pro-inflammatory form of programmed cell death, pyroptosis has accumulated abundant research findings regarding its basic mechanisms and disease associations. However, traditional research methods generally take a specific cell population or tissue as the research unit, treating the research object as a homogeneous whole. These methods conduct studies by detecting indicators such as the average population expression of pyroptosis-related molecules and the overall activity of related pathways (18–23), which not only overlook the response differences at the single-cell level but also fail to distinguish the heterogeneous characteristics of different cell subtypes in terms of pyroptosis activation efficiency and molecular mechanisms. Consequently, the occurrence and regulation of pyroptosis have been simplified into a homogeneous process. The application of scRNA-seq has overcome this limitation. By analyzing gene expression profiles at the single-cell level, it clearly reveals the significant transcriptional heterogeneity of pyroptosis-related genes among different cell subtypes and in various disease contexts, thereby supporting the concept that pyroptotic activity is not a homogeneous population behavior but a dynamic process with high cell subtype specificity and state dependence (**Table 1**). This understanding of heterogeneity based on single-cell resolution provides a crucial perspective for comprehending disease mechanisms and developing precise therapeutic strategies.

**Table 1:** Heterogeneity of pyroptosis in different cell subtypes and diseases.

Disease/model	Cell subtype	Species/model & sample size	Data type & dataset	Pyroptosis-inference method/gene set	Orthogonal/functional validation	Independent-cohort validation	Evidence level	Pyroptotic characteristics	Clinical and translational implication	Ref
Neoplastic diseases										
Melanoma	Immune cells (CD8^+^ T cells and NK cells)	Human; 9 in-house patient samples; bulk total 1560 samples; scRNA-seq sample count not stated in original paper	scRNA‐seq (GEO: GSE108394, GSE215120, GSE180885); bulk RNA‐seq (TCGA-SKCM, GTEx)	PRG gene set: GZMA, GSDMB, NLRP1, IL18, CHMP4A	IHC, multiplex‐IF, ELISA protein detection	No	T+P+R	Pyroptotic cell proportion: tumor tissue < normal skin tissues	Tumors weaken anti-tumor immunity by reducing pyroptosis-related immune cell infiltration	(24)
Melanoma	Immune cells (T cells, monocytes, and B cells)	Human; bulk total 1388 samples; scRNA-seq total 52 patients	Bulk RNA-seq (TCGA-SKCM, multiple GEO cohorts); scRNA-seq (GSE72056, GSE115978); spatial transcriptome (BayesSpace); partial proteomic datasets	73 curated PRGs; PScore model built on 31 prognostic-protective PRGs	partial proteome correlation analysis	Yes (8 fully non‐overlapping external cohorts)	T+R	PScore: T cells/monocytes/B cells > malignant cells/fibroblasts; pyroptosis-related transcriptional signatures upregulated in immune cell-infiltrated tumor boundary	Tumor boundary drives local inflammation and immune activation via pyroptosis	(25)
LUAD	Immune cells (MCs)	Human; in-house scRNA-seq:24 patients; integrated scRNA-seq total 115 samples; bulk total 1184 patient samples; *in vitro* mast-cell line	In-house plus public scRNA-seq; bulk RNA-seq (FUSCC_1019, FUSCC_150, OAK)	Canonical NLRP3-inflammasome/GSDMD-centered pyroptosis gene set; WB detection of cleaved GSDMD-N; PI staining, LDH-release assay, FLICA-Caspase-1 assay for mast-cell pyroptosis phenotyping	Multiplex‐IF, WB, imaging flow cytometry; CRISPR‐GSDMD KO, NLRP3/Caspase‐1 inhibition; LDH‐release, IL‐18‐ELISA pyroptosis assays	Yes (3 non‐overlapping patient cohorts)	T+P+F+R	Nine distinct MC functional states identified; proinflammatory MC subset (high IL-18) is the core mediator of pyroptosis-related functions	Proinflammatory MC subset infiltration correlates with favorable prognosis in LUAD patients	(27)
Liver cancer	Immune cells (monocytic-macrophages)	Human; bulk total 421 samples; scRNA-seq total 9 patient samples; *in vitro* THLE-2, Huh7 human liver cell lines	Bulk RNA-seq (TCGA-LIHC, GSE14520, HCCDB18); public scRNA-seq (GSE125449)	REACTOME_PYROPTOSIS gene set; GSVA for pyroptosis score calculation	qPCR mRNA measurement; Transwell/scratch-wound migration-invasion assays	Yes (2 non‐overlapping external cohorts)	T+P+F+R	Monocytic-macrophages exhibit the highest pyroptosis score	Provide new indicators for prognostic evaluation of liver cancer patients	(28)
Gliomas	Immune cells (BMDMs)	Human; bulk total 903 patient samples; scRNA-seq total 51 patients; in-house tissue microarray:114 samples	Bulk RNA-seq (TCGA, CGGA); public scRNA-seq datasets(GSE131928, GSE117891, GSE89567)	NLRC4/CASP1/CASP4/GSDMD gene-set	Tissue microarray IHC for GSDMD‐N and NLRC4 protein	Yes (TCGA as training cohort, CGGA as external validation cohort)	T+P+R	High-grade gliomas: BMDMs ↑ pyroptosis genes ↑	Pyroptosis is involved in tumor progression and immunosuppression	(29)
AAA	Immune cells (IFNICs)	Mouse: AAA disease model; Human: clinical aortic tissues (exact count not reported); *in vitro* THP-1 cell assays	Mouse-derived scRNA-seq dataset (GSE239620); human aortic tissue samples	Interferon-response gene set combined with canonical pyroptosis-related genes; WB detection of GSDMD, NLRP3, Caspase-1	*In vivo* myeloid-specific Sting1/Ifnar1 KO/bone-marrow transplant; THP-1 IRF7 siRNA knockdown; WB, IF for NLRP3, Caspase-1, GSDMD	No	T+P+F+A	Pyroptosis- and inflammation-related signaling pathways are specifically activated in IFNICs, regulated by IRF7	IFNICs are the key cellular target for disease progression	(30)
UCEC	Immune cells and non-immune cells (epithelial cells and macrophages)	Human; in-house clinical:42 patient tissues; bulk total 401 samples; scRNA-seq: 2 in-house samples	Self-generated scRNA-seq (ArrayExpress: E-MTAB-18643); public TCGA-UCEC; public GEO scRNA-seq datasets	Curated 51 canonical pyroptosis-related gene collection; AddModuleScore for pyroptosis signature scoring	Human-tissue RT-qPCR and IHC protein detection	No	T+P+R	Epithelial cells: high IL-6 expression disrupts epithelial proliferation balance, affects proliferation/apoptosis/cell communication Macrophages: high BAX/TNF expression dysregulates macrophage differentiation and immune responses	Heterogeneous pyroptosis gene expression in epithelial cells and macrophages synergistically drives disease progression	(31)
Non-neoplastic diseases										
Sepsis	Immune cells (neutrophils)	Human; single-center in-house cohort: 68 sepsis patients; bulk-seq: 14 patients; scRNA-seq: 6 subjects; PBMC multi-omics assays	Self-generated bulk mRNA-seq & miRNA-seq; self-generated 10× Genomics scRNA-seq	Canonical cell-death-related gene collection (pyroptosis, necroptosis, apoptosis, ferroptosis, autophagy); WB for cleaved-GSDMD/Caspase-5; electron-microscopy pore morphology	WB for cleaved-GSDMD/Caspase-5; electron-microscopy pore morphology; Luminex-ELISA inflammatory mediators; flow-cytometry, qRT-PCR	No	T+P+R	Neutrophil pyroptosis may exacerbate inflammatory response	Neutrophil pyroptosis correlates with poor sepsis prognosis; targeting this process provides a potential early immunotherapy direction	(26)
UC	Non-immune cells (fibroblasts)	Human; public bulk & sc‐RNA seq datasets (exact count not reported); in‐house paraffin colon sections; *in vitro* CCD‐18Co fibroblast line	Bulk transcriptome dataset (GSE193677); scRNA-seq dataset (GSE231993)	APOL1-NLRP3-Caspase-1-GSDMD gene-set; WB for NLRP3/cleaved-Caspase-1/GSDMD; LDH-release, PI-staining pyroptosis assays	Colon-tissue IHC-IF co-localization; CCD-18Co APOL1 overexpression; WB for NLRP3/cleaved-Caspase-1/GSDMD; LDH-release, PI-staining pyroptosis assays	No	T+P+F	High APOL1 expression in fibroblasts induces pyroptosis by activating the NLRP3/Caspase-1/GSDMD pathway	Act as a key driving mechanism for intestinal inflammation	(32)
Aging	Non-immune cells (brown adipocytes)	Mouse; multiple-age-group C57BL/6J transgenic/WT mice; snRNA-seq for brown adipose tissue (exact count not reported); primary brown-adipocyte *in vitro* assays	Single-nucleus RNA-seq (GSE248816, GSE229999); bulk RNA-seq	Pyroptosis-related gene-set: Casp1, Casp11, Nlrp1b, Aim2, Il18; WB detection of cleaved Caspase-1; PI staining, LDH-release assay, HMGB1 release, Caspase-Glo activity assay for brown-adipocyte pyroptosis phenotyping	Brown-adipocyte-specific Stx4 KO/overexpression; *in vivo* VX-765 Caspase-1/11 inhibition; primary-cell Cre-KO/shRNA-KD; LDH-release, PI-staining, HMGB1-IF, IL-18-ELISA; WB & tissue-IF	No	T+P+F+A	Aging progression upregulates the proportion of Ucp1-low brown adipocytes and pyroptosis-related gene expression	Aging is closely associated with enhanced pyroptosis in brown adipocytes	(33)
Autoimmune diseases										
Psoriasis	Non-immune cells (keratinocytes, mainly S.B)	Human; public scRNA-seq dataset (exact count not reported)	scRNA-seq dataset (GSE151177)	Human pyroptosis-related gene-set (CASP1, CASP4, CASP6, CASP8, GSDMA, GSDMB, GSDMC, GSDMD, GSDME)	Only mRNA-level qPCR in cell lines	No	T+P	Pyroptosis-susceptibility:S.B > S.C, S.G, S.S	Pyroptosis-associated transcriptional signatures play a prominent role in skin disorders, with negligible effects in neurological and reproductive autoimmune diseases	(34)
Atopic dermatitis	Non-immune cells (keratinocytes)	Human; public scRNA-seq dataset (exact count not reported)	scRNA-seq dataset (GSE147424); THP-1 cell-line bulk RNA-seq		Only mRNA-level qPCR/bulk transcriptome in cell lines	No	T+P	A unique pyroptosis-susceptible keratinocyte subset with high GSDMC and GSDMD expression (GSDMC^hi^GSDMD^hi^) is identified		
Vitiligo	Non-immune cells (epidermal melanocytes)	Human; public scRNA-seq dataset (exact count not reported)	scRNA-seq dataset (GSA-PRJCA006797)		Only mRNA-level qPCR in cell lines	No	T+P	Pyroptosis may participate in melanocyte destruction		
SSc-ILD	Non-immune cells (alveolar type 1&2 cells, smooth muscle cells, club/goblet/basal cells, etc)	Human; public scRNA-seq dataset (exact count not reported)	scRNA-seq dataset (GSE128169)		No	No	T	Multiple cell types and Gasdermin family members are involved		
MS	Non-immune cells (brain cells)	Human; public snRNA-seq dataset (exact count not reported)	snRNA-seq dataset (GSE118257)		No	No	T	Weak pyroptosis-associated transcriptional signals		
EAO	Non-immune cells (generative cell)	Mouse; public scRNA-seq dataset (exact count not reported)	mouse-derived scRNA-seq datasets (GSM5563668, GSM5563669)	Mouse pyroptosis-related gene-set (Casp1, Casp11, Casp6, Casp8, Gsdma1-3, Gsdmc, Gsdmd, Gsdme)	No	No	T	Weak pyroptosis-associated transcriptional signals		
Autoimmune liver disease	Non-immune cells (hepatocytes)	Mouse; spontaneous autoimmune-hepatitis model plus littermate controls; scRNA-seq for hepatic lymphocytes; *in vitro* hepatocyte co-culture assays	Mouse-derived targeted scRNA-seq dataset (GSE199065)	Curated pyroptosis gene-set from MSigDB (Gsdmd, Casp1, Casp3, Il1b, Il18, Aim2, Nlrp3); WB detection of cleaved N-GSDMD and cleaved Caspase-1; PI staining, FLICA-Caspase-1 assay, live-cell confocal imaging, IL-1β release for hepatocyte pyroptosis phenotyping	*In vivo* anti-PD-1 CAR-T depletion; disulfiram GSDMD-inhibitor treatment; *in vitro* Gsdmd CRISPR-KO; primary-cell co-culture; perforin-1/granzyme-B stimulation; LDH-release, FLICA-Caspase-1/PI staining; liver-tissue IHC & WB for N-GSDMD, cleaved-Caspase-1, IL-1β	No	T+P+F+A	PD-1^+^CD8^+^ T cell-mediated hepatocyte pyroptosis	Promote progression of murine autoimmune liver disease	(35)

Ref, references; NK, natural killer; scRNA-seq, single-cell RNA sequencing; PRGs, pyroptosis-related genes; PScore, pyroptosis-related gene score; LUAD, lung adenocarcinoma; MCs, mast cells; BMDMs, bone marrow-derived macrophages; AAA, abdominal aortic aneurysm; IFNICs, interferon-inducible monocytes/macrophages; UCEC, uterine corpus endometrial carcinoma; UC, ulcerative colitis; S.B, stratum basale; S.C, stratum corneum; S.G, stratum granulosum; S.S, stratum spinosum; SSc-ILD, systemic sclerosis-associated interstitial lung disease; MS, multiple sclerosis; EAO, experimental autoimmune orchitis.

T, transcriptional association; P, protein and/or mRNA level validation; F, *in vitro* functional perturbation; A, animal-model *in vivo* evidence; O, patient-derived tissue/organoid validation; R, retrospective clinical association; Pr, prospective clinical evidence. Combined codes (e.g., T+P) are used for multiple-layer evidence.

Differences in functional properties and microenvironmental adaptability among various cell types lead to significant heterogeneity in the expression of pyroptosis-related genes, signaling pathways, and biological effects. Among them, immune cells are core participants in pyroptosis. In melanoma, Zhang et al. (24) demonstrated that CD8^+^ T cells and natural killer (NK) cells highly expressed pyroptosis-related genes (such as GZMA and GSDMB) and exhibited transcriptional signatures associated with pyroptosis in target cells, serving as potential key executors of anti-tumor immunity. However, the proportion of such immune cells in tumor tissues was significantly lower than that in normal skin tissues, suggesting that tumors may weaken the anti-tumor immune response by reducing the infiltration of pyroptosis-related immune cells, thereby evading immune surveillance. Another single-cell analysis of melanoma showed that the pyroptosis-related gene score (PScore) of T cells, monocytes, and B cells was significantly higher than that of malignant cells and fibroblasts. Moreover, transcriptional signatures associated with pyroptosis were more prominent in the immune cell-infiltrated areas at the tumor boundary, suggesting that these cells may participate in local inflammatory responses and immune activation through pyroptosis (25). A scRNA-seq study on peripheral blood mononuclear cells (PBMCs) from sepsis patients showed that transcriptional signatures associated with immune cell death in the early stage of sepsis mainly occurred in myeloid cells (neutrophils and monocytes) rather than lymphocytes (26). Among these, neutrophils predominantly exhibited transcriptional signatures associated with destructive necrosis and pyroptosis. Transcriptional signatures of neutrophil pyroptosis were associated with exacerbated inflammatory response and poor prognosis in sepsis patients, suggesting that targeting neutrophil pyroptosis may provide a direction for early immunotherapy of sepsis (26). In lung adenocarcinoma (LUAD), Fan et al. (27) conducted an in-depth exploration of the pyroptosis heterogeneity of infiltrating mast cells (MCs) using single-cell sequencing technology. They identified 9 distinct functional states of MCs, among which the proinflammatory MC subset (with high IL-18 expression) represented the candidate population associated with pyroptosis-related transcriptional programs and exhibited significant differences from other MC subsets in the expression of pyroptosis-related molecules and inflammasome assembly activity. Meanwhile, this proinflammatory MC subset showed a higher infiltration level in indolent LUAD, decreased as the tumor progresses to the invasive stage, and its infiltration was associated with a favorable prognosis in LUAD patients. These findings not only reveal the molecular characteristics and functional differences of MC pyroptosis heterogeneity in LUAD but also provide a potential strategy for targeting the proinflammatory MC subset in LUAD immunotherapy.

Notably, most studies suggest that macrophages are most closely associated with pyroptosis, and single-cell sequencing has revealed that the pyroptotic heterogeneity of macrophages is reflected in their cellular origin and disease contexts. In liver cancer, it was reported that monocytic-macrophages exhibited the highest pyroptosis score and represented the core cell population in pyroptosis-related risk models, which helped provide new indicators for the prognostic evaluation of liver cancer patients (28). In gliomas, Li et al. (29) found that the tumor-infiltrating macrophages, particularly bone marrow-derived macrophages (BMDMs), rather than tissue-resident microglia, showed significant co-expression of GSDMD and CASP genes. Moreover, in high-grade gliomas, BMDMs accounted for a higher proportion of immune infiltrating cells and had higher expression of pyroptosis genes, suggesting that their pyroptosis may be involved in tumor progression and immunosuppression. In abdominal aortic aneurysm (AAA), Le et al. (30) also identified a subset of interferon-inducible monocytes/macrophages (IFNICs) using scRNA-seq. These were monocytes/macrophages that expressed interferon-stimulated genes (ISGs, such as IFIT3, ISG15, RSAD2, and IRF7), existed in a transitional state between monocytes and macrophages, and were terminally differentiated cells. They found that pyroptosis- and inflammation-related signaling pathways were specifically activated in IFNICs, and this process was regulated by IRF7, making IFNICs a key cellular target for disease progression.

If the pyroptosis of immune cells is closely associated with immune activation or suppression, then the pyroptosis of non-immune cells is more related to tissue specificity and pathological processes. In uterine corpus endometrial carcinoma (UCEC), Guo et al. (31) identified, via scRNA-Seq datasets, that pyroptosis-related genes showed heterogeneous expression in epithelial cells and macrophages of UCEC. Among them, epithelial cells, as the most abundant cell population in UCEC, accumulated a large number of pyroptosis-related genes, with the key gene IL-6 specifically upregulated in such cells. The abnormally high expression of IL-6 was predicted to disrupt the proliferation balance of epithelial cells, interfere with the specific regulation of ligand-receptor pairs in epithelial cells, and thereby potentially participated in tumorigenesis by affecting cell proliferation, apoptosis, and cell-cell communication. Meanwhile, the pyroptosis-related genes BAX and TNF were highly expressed in macrophages, and their abnormal high expression could lead to dysregulation of macrophage differentiation and immune responses. It is evident that the heterogeneous expression of pyroptosis genes in epithelial cells and macrophages does not exist in isolation but acts together to influence the progression of UCEC. In ulcerative colitis (UC), colonic fibroblasts highly expressed APOL1 and CXCL1, and single-cell sequencing revealed a co-localization of these two genes in fibroblasts (32). Overexpression of APOL1 could induce pyroptosis by activating the NLRP3/Caspase-1/GSDMD pathway, releasing inflammatory factors and chemokines, thus serving as an important driving mechanism for intestinal inflammation (32). This discovery revealed a new mechanism related to fibroblast pyroptosis in the pathogenesis of UC, providing a new perspective for understanding the development of UC. In addition to the aforementioned non-immune cells, Yu et al. (33) identified a specific cell population with pyroptotic characteristics in brown adipose tissue of aging through single-nuclei RNA sequencing, namely Ucp1-low adipocyte cell population. During the aging process, the proportion of these cells increased significantly, and the expression levels of pyroptosis-related genes (such as Casp1, Casp11, Nlrp1b, etc.) were synchronously upregulated, indicating a close association between aging and enhanced pyroptosis in brown adipocytes. Further studies have confirmed that the deletion of syntaxin 4 (STX4) was a key mechanism for activating the aforementioned pyroptotic signaling pathways, and this finding revealed the molecular link between aging, abnormal energy metabolism, and pyroptosis.

Pyroptosis exhibits distinct cell subset-specific involvement patterns not only in neoplastic and non-neoplastic diseases but also in autoimmune diseases. Through single-cell transcriptome analysis, Zhang et al. (34) revealed abnormal pyroptosis-related transcriptional signatures and significant heterogeneity across various autoimmune diseases, which was reflected in the differences in affected cell types, key molecules, and pathological contributions specific to different tissues. For instance, in psoriasis, there was an imbalance in pyroptosis sensitivity among different lineages of keratinocytes: the stratum basale (S.B) displayed transcriptional signatures suggestive of pyroptosis susceptibility, while the stratum corneum (S.C) and stratum granulosum/stratum spinosum (S.G&S.S) had low pyroptosis sensitivity. In atopic dermatitis, there existed a subset of pyroptosis-sensitive keratinocytes with high expression of GSDMC and GSDMD (GSDMC^hi^GSDMD^hi^). In vitiligo, epidermal melanocytes displayed the most prominent transcriptional signatures associated with pyroptosis. However, systemic sclerosis-associated interstitial lung disease (SSc-ILD) involved pyroptosis in multiple cell types, such as alveolar type 1&2 cells, smooth muscle cells, club/goblet/basal cells, etc. This cell type-specificity of pyroptosis reflects the regulatory role of the tumor microenvironment in cell sensitivity to pyroptosis. In terms of molecular mechanisms, there is heterogeneity in the key molecules relied on by pyroptosis in different autoimmune diseases: for example, atopic dermatitis mainly involved GSDMC/GSDMD, vitiligo primarily depended on GSDMD and CASP1/4, and SSc-ILD involved multiple members of the Gasdermin family (GSDMA/B/C/D) (34). Regarding the association with disease phenotypes, pyroptosis exerted a more prominent role in skin disorders (psoriasis, atopic dermatitis, vitiligo), whereas in neurological autoimmune diseases, such as multiple sclerosis (MS), its impact on brain cell destruction was weak and failed to act as a core driver; similarly, it played a minor role in reproductive system autoimmune diseases, such as experimental autoimmune orchitis (EAO) (34). These findings indicate that the pathological contribution of pyroptosis is closely related to tissue-specific inflammatory responses, providing a basis for precise targeted therapy against pyroptosis, which requires formulating strategies according to the heterogeneous characteristics of pyroptosis in different diseases. Another scRNA-seq study on a spontaneous severe murine model of autoimmune liver disease revealed that the occurrence of hepatocyte pyroptosis was regulated by specific cell subsets (35). They found that PD-1^+^CD8^+^ T cells, which exhibited specific expansion in the liver, were the key pathogenic cells of this disease (35). These cells induced gasdermin D (GSDMD)-dependent pyroptosis in hepatocytes by secreting high levels of granzyme B and perforin-1 (35). Depletion of PD-1^+^CD8^+^ T cells using anti-PD-1 chimeric antigen receptor T (CAR-T) cells reduced the intrahepatic level of N-terminal GSDMD (N-GSDMD) and inhibited the activation of pyroptosis-related pathways (35). This further confirms that hepatocyte pyroptosis induced by PD-1^+^CD8^+^ T cells is a core mechanism driving the progression of autoimmune liver disease, providing potential targets for the development of therapeutic strategies against this disease.

## 3 Pyroptosis-mediated microenvironment remodeling

In the microenvironment of tumors and other diseases, immune cells, parenchymal cells, stromal cells, and other components undergo changes in quantity, phenotype, or function. Meanwhile, the imbalanced transmission of intercellular communication signals (such as inflammatory factors and growth factors), the disruption of structural support systems (such as abnormal deposition or degradation of the extracellular matrix), and the remodeling of functional networks (such as disordered angiogenesis and imbalanced immune homeostasis) collectively drive the remodeling of the microenvironment. In this process, inflammatory factors and damage-associated molecular patterns (DAMPs) released by pyroptotic cells are the core driving forces for the remodeling of the local microenvironment.

Traditional studies mostly rely on animal models, cell line experiments, or bulk tissue detection techniques (36–41). Although they can confirm the correlation between pyroptosis and microenvironmental abnormalities, and even clarify partial causal relationships through genetic intervention, this “macro-targeted” research model has inherent limitations: it either relies on population-averaged data to mask individual cell differences, or focuses on a single signaling pathway or cell type, making it difficult to fully reconstruct the complex network of multi-cell and multi-pathway interactions in the microenvironment. As a result, the precise mechanism by which pyroptosis regulates the microenvironment has not been fully elucidated.

However, the combination of scRNA-seq and traditional research methods makes up for this deficiency: the “pyroptosis-microenvironment” correlation framework established by traditional studies provides a clear research direction and verification basis for scRNA-seq; by virtue of its single-cell resolution advantage, scRNA-seq can predict the source cells, target cells, and transmission pathways of pyroptotic signals through analyzing intercellular communication networks, and provide hypothesis-generating evidence for the molecular mechanism of dynamic interaction between pyroptosis and the microenvironment (**Table 2**). This complementary research model of “macro correlation-fine analysis” provides key technical support for holistically understanding the laws of disease microenvironment remodeling and developing pyroptosis-targeted microenvironment regulation strategies.

**Table 2:** Pyroptosis-mediated microenvironment remodeling.

Microenvironment remodeling category	Disease/model	Species/model & sample size	Data type & dataset	Pyroptosis-inference method/gene set	Orthogonal/functional validation	Independent-cohort validation	Evidence level	Microenvironmental characteristics and functional outcome	Ref
Immune microenvironment remodeling									
Pro-inflammatory immune microenvironment	UC	Human: bulk total 106 samples; scRNA-seq: 11 patients; mouse: 18 experimental mice	Bulk transcriptome (GSE87466, GSE92415, GSE107499, GSE59071, GSE73661, GSE46451); scRNA-seq (GSE162335)	PRG gene-set (UniProt/MSigDB)	Mouse-tissue qPCR, serum-ELISA, histopathology	No	T+P+A+R	Intestinal immune microenvironment pro-inflammatory polarization	(42)
	LN	Human: bulk total 47 samples; scRNA-seq:34 subjects; mouse: total 24 experimental mice; *in vitro* rat renal macrophage-tubular epithelial co-culture system	Bulk microarray (GSE32592, GSE104948); public scRNA-seq dataset	NLRP3-inflammasome-centered pyroptosis-related gene set; WB detection of cleaved-Caspase-1 and cleaved-IL-1β; ELISA measurement of IL-1β/IL-18 release	*In vivo* AAV-shRNA-NLRP3 gene knockdown in mice; *in vitro* NLRP3 overexpression/IL-33 siRNA knockdown in rat renal macrophages; WB, ELISA, immunofluorescence for pyroptosis-related proteins	No	T+P+F+A+R	Renal immune microenvironment destruction, leading to structural damage and functional failure	(43)
	Atherosclerosis	Human: vascular tissue samples; mouse: multiple groups of ApoE^-^/^-^ & Gsdmd^-^/^-^ApoE^-^/^-^ mice; *in vitro* mouse primary peritoneal macrophage experiments	Mouse aortic scRNA-seq	GSDMD-centered pyroptosis-related gene set; WB detection of cleaved GSDMD-N fragment; IHC for GSDMD tissue localization; ELISA measurement of downstream IL-1β/IL-18 release	*In vivo* Gsdmd knockout, bone-marrow transplantation, AAV-mediated macrophage-specific GSDMD knockdown, GSDMD-targeted pharmacological inhibition; *in vitro* Gsdmd-knockout primary macrophage functional assays; WB, immunofluorescence, ELISA for pyroptosis-associated proteins & inflammatory cytokines	No	T+P+F+A	Increased inflammatory factor release, enhanced macrophage migration, and aggravated local inflammatory infiltration	(44)
	Viral infection-associated metabolic diseases	Human; autopsy pancreatic samples (7 COVID-19, 8 control); human islet samples; hPSC-derived VMI organoid *in vitro* model	GeoMx spatial multi-omics; scRNA-seq & snATAC-seq(GSE247809)	Pyroptosis-related gene panel; immunofluorescence detection of cleaved-Caspase-1 in INS^+^ β-cells; ELISA measurement of IL-1β release	Human pancreatic-tissue immunofluorescence detection of CASP1; SARS-CoV-2/CVB4 virus infection and TNFSF12-/TNFRSF12A-targeted neutralizing-antibody intervention in VMI organoids	No	T+P+F+O	Pancreatic β cell damage and immune microenvironment pro-inflammatory remodeling	(45)
Immune-suppressive microenvironment	ccRCC	Human; bulk total >900 ccRCC patient samples; in-house clinical tissues: 16 paired tumor-paracancer for qPCR, 35 paraffin samples for IHC; ccRCC cell-line *in vitro* assays	TCGA-KIRC; multiple GEO/ArrayExpress/immunotherapy clinical cohorts; scRNA-seq	Prognostic pyroptosis-related gene panel; WB detection of GSDMB	Human clinical-tissue IHC/qPCR; ccRCC cell-line WB for GSDMB	Yes (validated in E-MTAB-1980, CheckMate-025, CheckMate-010 cohorts)	T+P+R	Immune-suppressive microenvironment formation, impairing anti-tumor immune response	(46)
Dynamic imbalance of immune microenvironment	Ovarian aging	Human; scRNA-seq:7 ovarian patient samples; 347 retrospective clinical subjects; *in vitro* THP-1 and human primary ovarian stromal-cell assays; mouse: multiple-age WT and GSDMD-KO mice	In-house human ovarian scRNA-seq	MSigDB-derived pyroptosis gene-set; WB detection of GSDMD-FL/cleaved GSDMD-N, cleaved-Caspase-1	Human ovarian-tissue IHC; follicular-fluid flow cytometry & ELISA; THP-1 macrophage pyroptosis induction and stromal-cell co-culture assays; GSDMD-KO mouse model, *in vivo* disulfiram pharmacological intervention; WB, histopathological staining for pyroptosis-related molecules	No	T+P+F+A+R	Immune microenvironment shifts from immunoregulatory to pro-inflammatory with age, promoting stromal cell senescence and declining reproductive function	(47)
	Inflammatory response of the hematopoietic system	Mouse; scRNA-seq: IFNα-treated and control mice bone-marrow HSPC samples; *in vivo* flow-cytometry immune-cell abundance assays	In-house mouse bone-marrow HSPC scRNA-seq (GSE226824)	Mouse GO-derived pyroptosis gene-set	*In vivo* flow cytometry for immune-cell population abundance	No	T+P+A	Increased pyroptosis-associated transcriptional signals, reduced myeloid progenitor cells, disruption of the hematopoietic immune microenvironment homeostasis, and impairment of hematopoietic function	(48)
	SCI	Mouse; contusion-induced SCI-model and sham-operated mice; *in vitro* BV2 microglia cell OGD/R model	Public bulk-RNA-seq datasets (GSE166967, GSE171441, GSE185301, GSE196928); public scRNA-seq dataset	PRG collection merged from literature, MGI and MSigDB	*In vivo* spinal-cord-tissue and *in vitro* BV2 cell qPCR for pyroptosis-related mRNA	No	T+P+A	Transcriptional signatures associated with pyroptosis first intensifies and then weakens during injury progresses, promoting the transformation of homeostatic microglia to inflammatory microglia and affecting spinal cord repair	(49)
	Simulated microgravity and spaceflight	Human; healthy donor PBMC *in vitro* simulated-microgravity experiments; astronaut-derived spaceflight multi-omics samples; mouse spleen space-flight samples	Self-generated human PBMC sc/bulk RNA-seq; spaceflight public multi-omics datasets	Canonical pyroptosis pathway signatures	*In vitro* PBMC cytokine detection (ELISA/Luminex); flow-cytometry immune-cell phenotyping; super-resolution cytoskeleton-morphology observation	No	T+P	“Pro-inflammatory yet immune-suppressive” immune microenvironment imbalance, with abnormalities in cell composition, pathogen control, and inflammatory senescence phenotype	(50)
Stromal microenvironment remodeling									
Stromal cell function transition/ECM remodeling	Silicosis	Human: 6 lung tissue specimens; mouse: silica-induced silicosis model (WT/Gsdmd-KO); scRNA-seq performed on 2 mouse lung samples; *in vitro* primary macrophage/fibroblast co-culture assays	In-house mouse lung scRNA-seq	GSDMD-centered canonical/non-canonical pyroptosis gene-set; WB detection for GSDMD-N, Caspase-1/11, IL-1β; LDH-release, SEM pyroptotic morphology detection	Gsdmd-KO mouse *in vivo* genetic ablation; *in vitro* primary macrophage silica stimulation; LDH-release, SEM pyroptotic morphology detection; WB/IHC-IF for GSDMD-N, Caspase-1/11, IL-1β; macrophage-fibroblast co-culture; pyroptotic-macrophage-derived exosome *in vivo* intervention; WB, immunofluorescence for fibrosis markers	No	T+P+F+A	Fibroblast differentiation into myofibroblasts, promoting ECM deposition and pulmonary fibrosis	(51) (52)
	PDAC	Human: 53 PDAC patient clinical tissues and serum samples; scRNA-seq: 6 patient specimens; mouse: KPC spontaneous PDAC mouse model; *in vitro* PDAC cell-line and primary myCAF functional assays	Patient-derived scRNA-seq	NLRP3-Caspase1-GSDMD canonical pyroptosis gene-set; WB detection of NLRP3, Casp1-FL, cleaved-Casp1, GSDMD-FL, GSDMD-N, GSDMD-C, ASC; cell-status quantification (Live/non-pyroptotic/pyroptotic cell death); GSDMD-N/DAPI immunofluorescence; ELISA for IL-1β/IL-18 release	*In vitro* circERC1 knockdown/overexpression in PDAC cells; pharmacological inhibition of Caspase-1; LDH-release, TEM pyroptotic morphology detection; WB/IHC for pyroptosis- and ECM-related proteins; *in vivo* EV-mediated circERC1 delivery intervention in KPC mice	No	T+P+F+A+R	Stellate cell differentiation into myCAFs with high ECM molecule expression, mediating chemoresistance	(53)
Angiogenesis regulation	ccRCC	Human; bulk total >900 ccRCC patient samples; in-house clinical tissues: 16 paired tumor-paracancer for qPCR, 35 paraffin samples for IHC; ccRCC cell-line *in vitro* assays	TCGA-KIRC; multiple GEO/ArrayExpress/immunotherapy clinical cohorts; scRNA-seq	Prognostic pyroptosis-related gene panel; WB detection of GSDMB	Human clinical-tissue IHC/qPCR; ccRCC cell-line WB for GSDMB	Yes (validated in E-MTAB-1980, CheckMate-025, CheckMate-010 cohorts)	T+P+R	Endothelial cell enrichment and angiogenesis activation	(46)
	OIR	Mouse: WT and multiple gene-knockout OIR model mice; *in vitro* HUVEC hypoxic functional assays	Mouse retinal scRNA-seq (GSE173079)	NLRP12-ASC-Casp8-GSDMD non-canonical inflammasome pyroptosis gene-set; WB detection of CASP1, cleaved GSDMD-N, mature IL-1β, NLRP12, ASC, cleaved CASP8; NLRP12-ASC-CASP8 inflammasome assembly confirmed by co-IP and dual immunofluorescence	*In vitro* siRNA-mediated gene knockdown/overexpression in hypoxic HUVECs; tube-formation functional assay; WB/co-IP/IF for inflammasome-pyroptosis-VEGF axis; *in vivo* GSDMD-KO, NLRP12-KO, Casp8-heterozygous knockout OIR mouse model retinal phenotype assessment	No	T+P+F+A	Release of VEGF and IL-1β, formation of retinal neovascularization, vascular leakage, and disruption of the ocular stromal microenvironment homeostasis	(54)

Ref, references; UC, ulcerative colitis; scRNA-seq, single-cell RNA sequencing; PRG, pyroptosis-related gene; LN, lupus nephritis; VMI, vascularized macrophage-islet; WT, wild type; ccRCC, clear cell renal cell carcinoma; SCI, spinal cord injury; ECM, extracellular matrix; TEM, transmission electron microscope; PDAC, pancreatic ductal adenocarcinoma; myCAFs, myofibroblastic cancer-associated fibroblasts; OIR, oxygen-induced retinopathy.

T, transcriptional association; P, protein and/or mRNA level validation; F, *in vitro* functional perturbation; A, animal-model *in vivo* evidence; O, patient-derived tissue/organoid validation; R, retrospective clinical association; Pr, prospective clinical evidence. Combined codes (e.g., T+P) are used for multiple-layer evidence.

### 3.1 Remodeling of the immune microenvironment

In various diseases, pyroptosis significantly enhances local immune responses and disrupts immune homeostasis by activating immune cells (especially macrophages) and releasing proinflammatory factors. In UC, scRNA-seq analysis revealed that macrophages exhibited transcriptional signatures indicating activation of pyroptosis-related pathways, with IL1B as a core signal. These macrophages could further differentiate into three subtypes: immunomodulatory macrophages, response macrophages, and transporter and secretory macrophages (42). These subtypes were associated with proinflammatory polarization of the intestinal immune microenvironment, putatively via enhancing inflammatory signals, transmitting stimulus responses, and spreading inflammatory factors, respectively (42). This finding not only clarifies the key role of macrophages in the remodeling of the UC colonic immune microenvironment through the pyroptotic pathway, but also confirms that IL1B is a core biomarker closely associated with pyroptosis in UC. A similar phenomenon has also been observed in another inflammatory disease, lupus nephritis (LN). In the renal tissue-resident macrophage subset (CM2 cells), the pyroptotic pathway was significantly activated. These macrophages mediated abnormal crosstalk with renal tubular epithelial cells via the NLRP3/IL-33/ST2/NF-κB axis, which activated the inflammatory response in epithelial cells, disrupted the renal immune microenvironment, and ultimately led to renal structural damage and functional failure (43). Honokiol (HNK) was found to directly bind to and inhibit NLRP3, thereby blocking the activation of the pyroptotic pathway (43). This reversal of LN-associated renal microenvironment remodeling provides a novel strategy for the treatment of LN. Fan et al. also reported another mechanism by which macrophages promote inflammation (44). During the development of atherosclerosis, GSDMD was mainly expressed in atherosclerotic macrophages, especially M1 macrophages (44). It caused mitochondrial perforation and mtDNA leakage, activated the STING/IRF3/NF-κB axis, and promoted the release of inflammatory factors and macrophage migration (44). This discovery reveals a new mechanism of atherosclerosis and provides a potential new target for the treatment of atherosclerosis. The pro-inflammatory effect mediated by pyroptosis also plays a crucial regulatory role in virus infection-related metabolic diseases. Using scRNA-seq, Yang et al. (45) found that after human islets were infected with SARS-CoV-2 or CVB4 virus, proinflammatory macrophages were activated (with high expression of proinflammatory genes such as IL1B and IL6), and the pyroptosis pathway in pancreatic islet β cells was significantly activated. Pyroptosis occurred in β cells regardless of whether they were directly infected by the virus. Meanwhile, the communication of the TNFSF12/TNFRSF12A signaling pathway between proinflammatory macrophages and β cells was enhanced, which, together with IL-1β, jointly mediates β cell pyroptosis and promoted the remodeling of the immune microenvironment toward a pro-inflammatory direction. This finding not only reveals a new mechanism by which proinflammatory macrophages damage pancreatic islet β cells through the pyroptosis pathway under viral infection but also provides a key theoretical basis for understanding the pathogenesis of COVID-19-related diabetes and virus-induced immune-mediated pancreatic islet damage.

In addition to its pro-inflammatory effects, pyroptosis can also shape an immunosuppressive microenvironment by regulating the infiltration and function of immune cells, thereby influencing the efficacy of tumor immunotherapy. In clear cell renal cell carcinoma (ccRCC), Ma et al. identified a pyroptosis-related cluster corresponding to an “Inflamed Phenotype” (46). Although immune pathways were activated in this subtype, it was accompanied by extensive infiltration of regulatory T cells (Tregs), exhausted CD8^+^ T cells, and cancer-associated fibroblasts, consistent with an immunosuppressive microenvironment phenotype (46). At the single-cell level, further investigation revealed significant heterogeneity in CD8^+^ T cells within the “Inflamed Phenotype”; most of these cells highly expressed exhaustion markers such as TIGIT and LAG3, and their exhausted state was associated with tumor stage (46). Meanwhile, the abnormal expression of cytokines (e.g., IL-4, IL-10, CCL5) in this subtype further exacerbated immunosuppression (46). This immunosuppressive microenvironment impaired the anti-tumor immune response, however, patients with this subtype exhibited a better response to PD-1 inhibitors (e.g., nivolumab), suggesting that the immunosuppressive microenvironment mediated by pyroptosis could be reversed through immune checkpoint inhibitors (46).

Under physiological aging, inflammatory stimulation, and special environmental conditions, pyroptosis can drive dynamic imbalance of the immune microenvironment, affecting tissue function and cellular homeostasis. Single-cell atlas of human ovaries revealed that during ovarian aging, transcriptional signatures of pyroptosis-related pathways in ovarian macrophages gradually increased with age, which promoted the dynamic transition of the ovarian immune microenvironment from an immunoregulatory type to a pro-inflammatory type (47). Pyroptotic macrophages further accelerated the senescence of stromal cells, leading to the decline of reproductive function (47). This finding reveals a novel mechanism of ovarian aging. In the hematopoietic system’s response to inflammation, single-cell time series analysis revealed that stimulation by interferon-alpha (IFNα) induced the upregulated expression of pyroptosis-related genes (Casp1, Casp4) in myeloid progenitors of the bone marrow (48). This led to an increase in the rate of pyroptosis, resulting in a sustained reduction in the number of myeloid progenitors and disruption of cellular homeostasis in the hematopoietic immune microenvironment (48). This dynamic change not only impaired hematopoietic function but also might correlate with the pathogenesis of autoimmune diseases (e.g., systemic lupus erythematosus). In spinal cord injury (SCI), Li et al. (49) found through scRNA-seq that transcriptional signatures associated with pyroptosis first intensified and then weakened as the injury progresses. Transcriptional pyroptosis-related responses reached a peak at 7 days post SCI. A core change in the immune microenvironment was the transformation of homeostatic microglia into inflammatory microglia, and the pyroptosis of inflammatory microglia was positively correlated with the infiltration of macrophages and monocytes. These findings clarify the dynamic characteristics of the immune microenvironment mediated by pyroptosis after SCI, and provide an important theoretical basis for the precise regulation of pyroptosis to alleviate inflammation and promote spinal cord repair. In some special environments, pyroptosis can also mediate immune imbalance. Through scRNA-seq analysis, Wu et al. (50) found that in simulated microgravity and spaceflight, the pyroptotic pathway was abnormally regulated in immune cells: the pyroptotic pathway in innate immune cells such as monocytes and B cells was suppressed, and the suppression of pyroptosis in T cells and NK cells was further exacerbated after stimulation with TLR7/8. This may prevent immune cells from normally initiating pyroptosis-mediated inflammatory alert and immune clearance functions. This pyroptotic imbalance triggered multi-dimensional disorders in the immune microenvironment, including abnormal monocyte function, impaired T/NK cell function, imbalanced cell composition with increased Treg infiltration, and reduced pathogen control ability due to the transcriptional activation of latent pathogens. Collectively, these changes are consistent with imbalanced microenvironment state of “pro-inflammatory yet immunosuppressive”.

### 3.2 Remodeling of the stromal microenvironment

The stromal microenvironment is a crucial component of the microenvironment, centered on acellular components [extracellular matrix (ECM), cytokines, metabolites] and stromal cells. It supports cells through physical structures and regulates cell survival, proliferation, and functional states via signal transduction, playing a decisive role in tumor progression and the pathological processes of infectious diseases. Inflammatory mediators released by pyroptosis can target and regulate the components of the stromal microenvironment through cascade signal transduction: inducing functional transitions of stromal cells (such as fibroblast activation), driving ECM remodeling, and regulating angiogenesis, ultimately achieving pathological remodeling of the stromal microenvironment. This regulatory mechanism provides a new perspective for in-depth analysis of the rules governing disease occurrence and development, as well as the development of targeted therapeutic strategies.

Silicosis is an occupational lung disease characterized by progressive pulmonary fibrosis, for which there are no effective treatments. Recent studies have found that silica (or silica nanoparticles) could induce GSDMD-dependent pyroptosis in macrophages in silicosis. This process was co-driven by both the canonical pathway (mediated by caspase-1) and the non-canonical pathway (mediated by caspase-4/5/11) (51, 52). On one hand, pyroptotic macrophages released pro-inflammatory factors such as IL-1β, which directly exacerbated pulmonary inflammation and collagen deposition (51). On the other hand, they secreted exosomes enriched with specific microRNAs (miRNAs), such as miR-21a-5p and miR-877-3p (52). After being taken up by fibroblasts, these exosomes promoted the differentiation of fibroblasts into myofibroblasts by regulating signaling pathways like the transforming growth factor-β (TGF-β) pathway, thereby driving ECM deposition and pulmonary fibrosis, and ultimately achieving pro-fibrotic remodeling of the stromal microenvironment (52). Notably, inhibiting GSDMD or related pyroptotic pathways could significantly alleviate the progression of silicosis. In addition to fibrotic diseases, pyroptosis-mediated remodeling of the stromal microenvironment also plays a crucial role in tumor chemoresistance. Zhang et al (53). performed scRNA-seq on tissue samples from pancreatic ductal adenocarcinoma (PDAC) patients who received gemcitabine combined with nab-paclitaxel (GEM-NabP) chemotherapy or direct surgery. They found that in the chemotherapy group, the number of fibroblasts in the TME increased and ECM-related pathways were enriched. Pseudotime analysis revealed that stellate cells differentiated into myofibroblastic cancer-associated fibroblasts (myCAFs) with high expression of ECM molecules. Further studies confirmed that after extracellular vesicles (EVs)-packaged circERC1 was taken up by myCAFs, it upregulated COL1A1 by sequestering miR-129-5p to enhance ECM deposition. Meanwhile, circERC1 bound to hnRNPA1, blocked its SUMOylation, promoted its degradation, and inhibited the pyroptosis pathway, ultimately mediating chemoresistance through this dual mechanism. This study clarified that circERC1 can serve as a biomarker for chemoresistance and poor prognosis in PDAC, and targeting circERC1 can regulate pyroptosis-mediated ECM remodeling to improve treatment outcomes.

In the regulation of tumor angiogenesis, the differential expression of pyroptosis-related genes also affects disease progression by remodeling the stromal microenvironment. Ma et al. (46) analyzed the tumor microenvironment of ccRCC using single-cell RNA sequencing (scRNA-seq) technology and found that clustering based on pyroptosis-related genes could classify ccRCC into two subtypes: the “Angiogenesis Phenotype” (Cluster 1) and the “Inflamed Phenotype” (Cluster 2). Among them, the “Angiogenesis Phenotype” exhibited significant angiogenesis activation characteristics during stromal microenvironment remodeling. scRNA-seq revealed that the tumor microenvironment of this subtype was enriched with endothelial cells, along with enrichment of classical oncogenic pathways such as mTOR, Notch, and VEGF, as well as angiogenesis-related pathways. Additionally, angiogenesis-related proteins including AKT pS473, VEGFR2, and CD31 were highly expressed. These findings indicate that pyroptosis-related gene signatures are associated with tumor angiogenesis, potentially via regulation of endothelial cell function in the stromal microenvironment and activation of relevant pathways. Meanwhile, it was found that patients with the “Angiogenesis Phenotype” showed a better response to anti-angiogenic therapies such as sunitinib, providing hypothesis-generating evidence for selecting personalized treatment regimens based on pyroptosis-related subtype characteristics in retrospective cohorts. The regulation of angiogenesis by pyroptosis-mediated stromal microenvironment remodeling is not limited to the field of oncology, a similar mechanism also exists in ocular pathological vascular diseases. Li et al. (54) discovered through scRNA-seq that in the oxygen-induced retinopathy (OIR) model, the downregulation of AIP1 expression in vascular endothelial cells (VECs) activated the non-canonical NLRP12/ASC/Caspase-8 inflammasome, which in turn triggered GSDMD-dependent pyroptosis of VECs, promoting the release of VEGF and IL-1β, exacerbating retinal neovascularization and vascular leakage, and disrupting the homeostasis of the ocular stromal microenvironment. Meanwhile, upregulating AIP1 or inhibiting the NLRP12/Caspase-8/GSDMD axis suppressed the VEGF signaling pathway, reduced pathological angiogenesis, and improved retinal vascular lesions in OIR mice. This finding reveals a novel mechanism by which pyroptosis-mediated stromal microenvironment remodeling regulates ocular angiogenesis, and provides potential therapeutic targets for activating AIP1 or inhibiting the pyroptosis pathway in pathological ocular angiogenesis diseases such as diabetic retinopathy and retinopathy of prematurity.

## 4 Prognosis-related pyroptosis gene signatures: insights from scRNA-seq

As a core pro-inflammatory programmed cell death mode, pyroptosis-related gene signatures (PRGS) have become a key source of biomarkers for disease prognosis assessment. Traditional prognostic models mostly rely on bulk RNA-seq (55–62), although this technology enables large-sample gene expression analysis, it is difficult to avoid the problem of information masking caused by microenvironment heterogeneity. In recent years, by integrating scRNA-seq with bulk RNA-seq (and spatial transcriptomics in some studies), it has become possible to more accurately screen core genes and construct pyroptosis-related gene signatures that not only possess clinical prognostic value but also reflect associations with the microenvironment, effectively compensating for the limitations of a single technology (**Table 3**). Different studies adopt distinct nomenclature for these prognostic models (e.g., PRGS, PScore, PRS). Fundamentally, they are quantitative prognostic evaluation models built on the expression levels of core pyroptosis-related genes. We discuss the applications of these models in specific diseases collectively in the following sections.

**Table 3:** Prognosis-related pyroptosis gene signatures: insights from scRNA-seq.

Disease/model	Species/model & sample size	Data type & dataset	Pyroptosis-inference method/gene set	Orthogonal/functional validation	Independent-cohort validation	Evidence level	Core PRGS genes	Signature type	Functional and clinical features	Ref
Tumor Diseases										
GC	Human: bulk transcriptome n=414; scRNA-seq n=6; clinical tissue microarray: 54 paired GC-adjacent specimens	TCGA-STAD; scRNA-seq (GSE183904); GSE62254	PRG collection merged from MSigDB and published literatures	IHC staining of core PRGS proteins	Yes (retrospective external validation in public cohort GSE62254)	T+P+R	C12orf75, VCAN, RGS2, MKNK2, SOCS3, TNFAIP2	Prognostic + Putatively predictive	• High-risk group: poor prognosis; immunosuppressive TME; computational inference indicates sensitivity to cisplatin • Low-risk group: computational inference indicates better responses to 5-fluorouracil and paclitaxel • VCAN is a key molecule shaping the immunosuppressive TME	(63)
CRC	Human: bulk transcriptome n=566; scRNA-seq n=5; *in vitro* normal colonic and CRC cell lines (NCM-460, HT-29, HCT116)	GSE39582; TCGA-COAD/READ; scRNA-seq (GSE132257)	PRG collection merged from MSigDB and published literatures	RT-qPCR detection of core PRGS mRNA expression *in vitro*	Yes (retrospective independent external validation in TCGA-COAD/READ cohort)	T+P+R	CHMP6, GSDMD, GZMB, NLRP1, CYCS, CASP3, CASP1, CASP6	Prognostic + Putatively predictive	• PRS correlates with pathological and AJCC-TNM stages, and may act as an independent prognostic indicator • Low-PRS group: better OS/PFS, high immune gene expression, abundant anti-tumor immune cell infiltration; computational inference indicates better response to 5-fluorouracil and anti-PD-1 immunotherapy	(64)
HCC	Human: bulk transcriptome n=335; scRNA-seq total n=53	TCGA-LIHC; ICGC-LIRI-JP; scRNA-seq (GSE149614, GSE156625); GSE91061, GSE78220, GSE109211, GSE104580	PRG collection derived from GeneCard database	No	Yes (retrospective independent external validation in ICGC-LIRI-JP cohort)	T+R	ERGIC3, STAT4, MGMT	Prognostic + Putatively predictive	• PR risk may be an independent prognostic indicator • High-PR risk group: high malignant cell proportion, strong cancer stem cell properties, oncogenic pathway activation, “cold tumor” phenotype, and poor prognosis • Low-PR risk group: abundant immune infiltration; computational inference indicates better response to ICBs, sorafenib and TACE; enhanced ligand-receptor pair communication	(65)
HCC	Human: bulk transcriptome n=337; scRNA-seq total n=18; HCC cell lines; clinical tissue specimens for IHC n=2	TCGA-LIHC; ICGC-LIRI-JP; scRNA-seq(GSE149614)	PRG collection merged from GeneCards, MSigDB and published literatures	RT-qPCR detection of core PRGS mRNA in multiple HCC cell lines; IHC staining of partial PRGS proteins in human HCC clinical tissues	Yes (retrospective independent external validation in ICGC-LIRI-JP cohort)	T+P+R	BAX, TREM2, CHMP4B, CHMP3, GBP1, IRF1, CHMP2A, MST1	Prognostic + Putatively predictive	• The risk score has a strong predictive ability for one-year, two-year, and three-year OS • High-risk group: decreased OS, low TIDE score, high PD-1/CTLA4 expression, computationally inferred higher immunotherapy sensitivity, increased M0 macrophage infiltration and decreased M1 macrophages • PRGS genes correlate with IC50 of paclitaxel and fluorouracil	(66)
Metastatic Melanoma	Human; bulk total 1388 samples; scRNA-seq total 52 patients	Bulk RNA-seq (TCGA-SKCM, multiple GEO cohorts); scRNA-seq (GSE72056, GSE115978); spatial transcriptome (BayesSpace); partial proteomic datasets	73 curated PRGs; PScore model built on 31 prognostic-protective PRGs	partial proteome correlation analysis	Yes (8 fully non‐overlapping external cohorts)	T+R	31 prognostic protective genes: AIM2, APIP, CASP1, CASP3, CASP4, CASP5, CASP8, CFLAR, CHMP2B, CHMP4A, CHMP5, DFNB59, GSDMB, GSDMD, GZMA, GZMB, IL18, IL1B, IRF1, IRF2, MEFV, NAIP, NLRC4, NLRP1, NLRP3, NLRP6, NLRP7, NOD2, TLR4, TNFRSF1B, ZBP1	Prognostic + Putatively predictive	• PScore may be an independent prognostic factor • High PScore: better survival; computationally inferred better ICB therapy efficacy • PScore correlates with BRAF mutations and is positively linked to immune-related pathways • Core genes are highly expressed in immune cells (highest in monocytes) • PScore in tumor boundary is higher than in tumor core and stromal regions	(25)
GBM	Human: bulk transcriptome n=246; scRNA-seq total n=15; *in vitro* GBM cell lines (SF-295, HS-683)	TCGA-GBM; CGGA; scRNA-seq(GSE141383, GSE223063); GSE13041, GSE74187, GSE83300; TCIA-IPS; GDSC2 drug-sensitivity database	PRG collection curated from published literatures; multi-algorithm pyroptosis scoring(AUCell, UCell, singscore, ssGSEA, AddModuleScore)	siRNA-mediated MAP1B knockdown in GBM cell lines; CCK-8, EdU, Transwell migration-invasion functional assays; RT-qPCR for knockdown efficiency validation	Yes (retrospective independent external validation in CGGA, GSE13041, GSE74187, GSE83300 cohorts)	T+P+F+R	PDLIM4, MAP1B, MGP, POSTN, LGALS1, CCL2, ANXA1, MYL12A, CLIC1, GSN	Prognostic + Putatively predictive	• Significant heterogeneity of pyroptosis-related transcriptional signatures: high in macrophages and T/B cells, low in tumor cells pyroptosis-related transcriptional signatures • High-PRGS scores: poor prognosis, elevated TIDE score (high immune-escape potential), low immunotherapy benefit (low immunophenoscore value), and enrichment of metabolic reprogramming and DNA repair pathways • MAP1B, as a key oncogene, promotes proliferation, migration, and invasion of GBM cells	(67)
ccRCC	Human: bulk transcriptome n=516; published public scRNA-seq dataset (exact count not reported)	TCGA-KIRC; GSE29609; public scRNA-seq dataset from published literature	PRG collection from published literatures	No	Yes (retrospective independent external validation in GSE29609 cohort)	T+R	CASP3, CASP4, GSDMB, GZMA	Prognostic	• High-risk group: short OS, immunosuppressive TME characteristics • Model genes are more highly expressed in cancer cells than in adjacent non-tumor tissues	(68)
Non-Tumor Diseases										
OA	Human: bulk transcriptome n=107; scRNA-seq total n=5	GSE12021, GSE55235, GSE82107, GSE89408; scRNA-seq(GSE169454)	PRG collection derived from published literatures	No	No	T	• 10 PRDEGs: CASP1, CASP3, CASP6, CASP8, GSDMB, NLRP1, NOD1, PLCG1, PRKACA, PYCARD; • 15 core hub genes: WAS, AGT, CTSD, APOE, CYBA, NCF1, ITGAM, AGTR1, CD74, CORO1A, ITGB2, PTPN6, THBS1, CD4, CD14	Prognostic	• Pyroptosis score enables prognostic stratification • Hub genes regulate ROS metabolism, immune infiltration, and chondrocyte metabolism, and form functional crosstalk with PRDEGs in pathways (phagosome/lysosome), immune microenvironment, and chondrocyte subtypes	(69)
OA + RA	Human: bulk microarray n=79; scRNA-seq dataset (total cells=25245); human synovial tissues for IHC (OA n=10, RA n=10); mouse DMM-induced OA model (sham n=6, DMM n=6)	GSE55235, GSE55457, GSE55584; scRNA-seq(GSE152805)	RNA-modification-related gene collection; WGCNA co-expression network for pyroptosis-phenotype-correlated module screening; intersection of module genes and RNA-modification-related DEGs	IHC staining on human OA/RA synovial tissues; histological staining and IHC for mouse DMM OA model	No	T+P+A	• 6 RNA modification-related disease-specific biomarkers: OA (OGN, TNFRSF11B, SCARA3, PTN), RA (ADAMDEC1, IGHM); • 6 pyroptosis-RNA modification associated hub genes: CXCL10, CXCL9, CCR7, CCL5, CXCL1, CCR2	Prognostic + Diagnostic	• Disease-specific biomarkers distinguish OA from RA • Hub genes clarify the synergistic regulatory mechanism of pyroptosis, RNA modification, and immune infiltration • Both gene sets jointly regulate inflammation and pyroptosis, affecting disease progression and prognosis	(70)
NEC	Human: bulk transcriptome; scRNA-seq n=11; human intestinal clinical tissues n=6; *in vitro* THP-1 macrophage cell line	GSE46619; scRNA-seq(GSE178088)	Curated pyroptosis-related gene collection from published resources; pyroptosis scoring; WB detection of cleaved-GSDMD-N, cleaved-Caspase-1, mature IL-1β; LDH-release assay	Immunofluorescence and qRT-PCR on human intestinal tissues; siRNA-mediated TREM1 knockdown in THP-1 macrophages; Western blot, qRT-PCR and LDH-release functional assays	No	T+P+F	TREM1, TLN1, NOTCH2, MPZL1, ADA	Prognostic + Diagnostic	• TREM1 is highly expressed in NEC intestinal tissue, activating pyroptosis to aggravate intestinal injury and adverse prognosis • TREM1^+^CD163^+^ macrophages are critical regulators of pyroptosis in NEC	(71)
T2D	Human: bulk islet transcriptome n=37; scRNA-seq n=11	GSE118139, GSE25724, GSE20966; scRNA-seq(GSE153855)	Curated pyroptosis-related gene collection from MSigDB and published literatures; GSVA pyroptosis scoring	No	No	T+R	• 3 genes for T2D onset risk prediction: GBP2, NLRP1, NOD2; • 17 pyroptosis-related hub genes: INS, CHGA, GCG, GAD2, NEUROD1, PCSK1, ABCC8, GRIA2, CHGB, STMN2, GNAS, IAPP, CPE, KCNQ1, NKX2-2, SCG5, SLC17A6	Prognostic + Diagnostic	• Risk prediction genes achieve accurate prediction of T2D onset risk • Hub genes reflect long-term pathological alterations of T2D, including islet dysfunction and immune microenvironment disturbance, supporting long-term prognostic evaluation	(72)
AD	Human: bulk brain transcriptome n=321; scRNA-seq n=12; human AD brain clinical specimens; APP/PS1-21 AD-model mice; *in vitro* BV2 microglial cell line	GSE138260, GSE173955, GSE118553; scRNA-seq(GSE188545)	Curated pyroptosis-related gene collection from MSigDB and published literatures; WB detection of N-GSDMD; LDH-release, ROS, JC-1 (mitochondrial membrane potential), IL-1β/IL-18 assays	WB and immunofluorescence on human AD brain and APP/PS1-21 mouse brain tissues; CHMP4B over-expression in BV2 microglia; qRT-PCR, WB, LDH-release, ROS and JC-1 mitochondrial membrane potential detection	Yes (retrospective independent external validation in GSE33000 and GSE44772)	T+P+F+A+R	GSDMD, CHMP4B, CHMP4A, CHMP2A, HMGB1, CASP3	Prognostic + Diagnostic	• CHMP4B expression is negatively correlated with AD severity, which can stratify patient prognosis • Gene combination provides high accuracy and specificity for the clinical diagnosis of patients with AD	(73)

Ref, references; scRNA-seq, single-cell RNA sequencing; PRGS, pyroptosis-related gene signatures; GC, gastric cancer; TME, tumor microenvironment; CRC, colorectal cancer; PRS, pyroptosis-related risk score; OS, overall survival; PFS, progression-free survival; HCC, hepatocellular carcinoma; PR, pyroptosis-related; ICBs, immune checkpoint blockades; TACE, transcatheter arterial chemoembolization; TIDE, tumor immune dysfunction and exclusion; PScore, pyroptosis-related score; GBM, glioblastoma; ccRCC, clear cell renal cell carcinoma; reactive oxygen species, ROS; OA, osteoarthritis; PRDEGs, pyroptosis-related differentially expressed genes; RA, rheumatoid arthritis; DMM, destabilization of the medial meniscus; NEC, necrotizing enterocolitis; T2D, Type 2 diabetes; AD, alzheimer’s disease.

T, transcriptional association, P, protein and/or mRNA level validation, F, *in vitro* functional perturbation, A, animal-model *in vivo* evidence, O, patient-derived tissue/organoid validation, R, retrospective clinical association, Pr, prospective clinical evidence. Combined codes (e.g., T+P) are used for multiple-layer evidence.

### 4.1 PRGS in tumor diseases

In digestive system tumors, Li et al. (63) analyzed GC samples via scRNA-seq and identified 9 pyroptosis-related genes (PRGs) with differential expression across different cell subpopulations, among which myeloid cells and monocytes exhibited higher PRG activity. Building on this finding, Li et al. further integrated bulk RNA-seq data to construct a PRGS comprising 6 core genes: C12orf75, VCAN, RGS2, MKNK2, SOCS3, and TNFAIP2. Risk scores derived from this signature effectively stratified patient prognosis: patients in the high-risk group had poorer prognosis and displayed a significantly immunosuppressive TME, characterized by increased infiltration of Tregs and M2-type tumor-associated macrophages (TAMs), elevated CD274 expression, and higher sensitivity to cisplatin. In contrast, patients in the low-risk group showed better responses to classical chemotherapeutic agents such as 5-fluorouracil and paclitaxel. Notably, within this signature, VCAN was highly expressed in GC tissues and positively correlated with Treg infiltration, M2 TAM infiltration, and CD274 expression, making it a key molecule in shaping the immunosuppressive TME. This gene signature not only provides a reliable prognostic tool for GC and hypothesis-generating transcriptomic evidence for potential drug-response associations, but also reveals the potential of VCAN as a target for immunotherapy. Similarly, in colorectal cancer (CRC), Ding et al. (64) adopted this multi-omics integration strategy to construct a PRGS containing 8 core genes (CHMP6, GSDMD, GZMB, NLRP1, CYCS, CASP3, CASP1, and CASP6) and calculated a pyroptosis-related risk score (PRS) based on this signature. CRC patients in the low-PRS group had significantly better overall survival (OS) and progression-free survival (PFS) than those in the high-PRS group. Additionally, the low-PRS group showed higher expression of immune-related genes, more abundant infiltration of anti-tumor immune cells (e.g., activated CD8^+^ T cells and activated CD4^+^ T cells), and greater sensitivity to 5-fluorouracil-based chemotherapy and anti-PD-1 immunotherapy. Single-cell analysis revealed that cell clusters with low pyroptosis-related gene expression activity, as quantified by the AUCell scoring algorithm (an AUC-based gene-set activity-recovery method for single-cell transcriptomes), were more abundant in the low-AUC group. This may partially explain differences in immunotherapy sensitivity across patients. PRS was also associated with CRC pathological stage and AJCC-TNM stage and served as an independent prognostic indicator. This signature provides a reliable prognostic biomarker for CRC, accurately stratifies patient populations based on treatment sensitivity, and provides hypothesis-generating transcriptomic evidence for potential drug-response associations with chemotherapy and immunotherapy.

In the field of hepatocellular carcinoma (HCC), Liu et al. (65) also explored the integration of scRNA-seq and bulk RNA-seq, constructing a PRGS based on three core genes (ERGIC3, STAT4, and MGMT) and establishing a corresponding PR risk scoring system. They found that PR risk was an independent prognostic indicator for HCC: patients with high PR risk had a higher proportion of malignant cells, stronger cancer stem cell properties, and more significant activation of oncogenic pathways. Moreover, their TME showed reduced infiltration of immune cells such as NK cells and effector CD8^+^ T cells, presenting a “cold tumor” phenotype and poorer prognosis. In contrast, patients with low PR risk had more abundant immune infiltration and better predicted responses to neoadjuvant therapies including immune checkpoint blockades (ICBs), sorafenib, and transcatheter arterial chemoembolization (TACE). Cellular communication analysis revealed enhanced signaling via ligand-receptor pairs such as HRAS/SDC2 and CDH1/IGF1R in high-risk patients, suggesting HRAS and IGF1R as potential prognostic targets. This gene signature not only provides an efficient tool for HCC prognostic stratification and treatment response prediction but also clarifies precise therapeutic directions for patients with different PR risks. Low-risk patients may receive neoadjuvant therapy, whereas high-risk patients are candidates for HRAS/IGF1R-targeted therapy, providing theoretical support and candidate directions for precision medicine in HCC.

Another HCC study, based on scRNA-seq analysis, identified 29 PRGs enriched in NK cells, T lymphocytes, monocytes, and macrophages of HCC tissues (66). Subsequent integration of bulk transcriptomic data led to the construction of a PRGS including 8 genes: BAX, TREM2, CHMP4B, CHMP3, GBP1, IRF1, CHMP2A, and MST1. The risk score had a strong predictive ability for one-year, two-year, and three-year OS. Based on PRGS, high-risk patients had decreased OS, lower Tumor Immune Dysfunction and Exclusion (TIDE) scores, elevated PD-1 and CTLA4 expression (suggestive of higher sensitivity to immunotherapy), and an immune microenvironment characterized by increased M0 macrophage infiltration and decreased M1 macrophage infiltration. Genes in the PRGS were significantly correlated with the half-maximal inhibitory concentration (IC50) of multiple drugs (e.g., paclitaxel and fluorouracil) and were abnormally upregulated in HCC cell lines and clinical tissues. This PRGS provides a reliable prognostic tool for HCC and offers molecular evidence for the precise selection of immunotherapies (e.g., PD-1/CTLA4 inhibitors) and targeted therapies.

In melanoma research, Chen et al. (25) integrated multi-omics technologies including bulk RNA-seq, scRNA-seq, and spatial transcriptomics to systematically investigate the prognostic value of pyroptosis in metastatic melanoma. First, the authors constructed a pyroptosis-related gene score (PScore) model based on bulk RNA-seq, which comprised 31 prognostic protective PRGs (AIM2, APIP, CASP1, CASP3, CASP4, CASP5, CASP8, CFLAR, CHMP2B, CHMP4A, CHMP5, DFNB59, GSDMB, GSDMD, GZMA, GZMB, IL18, IL1B, IRF1, IRF2, MEFV, NAIP, NLRC4, NLRP1, NLRP3, NLRP6, NLRP7, NOD2, TLR4, TNFRSF1B, and ZBP1). They found that PScore was an independent prognostic factor for metastatic melanoma, with patients exhibiting high PScore showing better survival outcomes. Additionally, PScore was associated with BRAF mutations and positively correlated with immune-related pathways such as the γ-interferon (IFN-γ) response and KRAS signaling. Single-cell analysis revealed that the 31 core genes were more actively expressed in immune cells, among which monocytes had the highest PScore, while malignant cells and fibroblasts had the lowest. Spatial transcriptomics further demonstrated that the PScore at the tumor border (a region infiltrated by macrophages and T/B cells) was significantly higher than that in the tumor core and stromal regions. Meanwhile, high PScore was associated with better efficacy of ICB therapy. This signature provides a prognostic biomarker for metastatic melanoma, enables prediction of immunotherapy response, and offers a theoretical basis for pyroptosis-related combination therapies (e.g., combination with BRAF-targeted therapy). Fang et al. (67) applied a similar multi-omics integration approach to the study of glioblastoma (GBM). By integrating scRNA-seq and bulk RNA-seq, combined with ten machine learning algorithms and their combinations, they constructed a PRGS. The core component genes of this signature included PDLIM4, MAP1B, MGP, POSTN, LGALS1, CCL2, ANXA1, MYL12A, CLIC1, and GSN. The study found that transcriptional signatures associated with pyroptosis activity was heterogeneous among GBM cell populations: macrophages and T/B cells exhibited the strongest transcriptional signatures associated with pyroptosis, while tumor cells showed relatively weak signatures. They also discovered that the SPP1-centered signaling axis was significantly remodeled in the high-pyroptosis group, mediating crosstalk between tumor cells and immune cells. Patients with high PRGS scores had poorer prognosis, accompanied by higher immune escape potential (increased TIDE score) and lower immunotherapy benefits (decreased immunophenoscore value). Furthermore, high PRGS scores were enriched in metabolic reprogramming and DNA repair pathways, whereas low PRGS scores were associated with immune activation, and there were computationally inferred differences in drug sensitivity between the two groups. As a key oncogene, MAP1B could promote the proliferation, migration, and invasion of GBM cells. This PRGS provides a reliable prognostic biomarker for GBM, enabling accurate risk stratification and prediction of immunotherapy response. It also reveals the molecular mechanisms of pyroptosis in tumor metabolism and immune regulation, and offers an important theoretical basis for the development of targeted therapy and precision immunotherapy strategies for GBM. PRGS also demonstrated clinical value in urinary system malignancies. Based on the TCGA database of ccRCC, Gan et al. (68) constructed a PRGS containing 4 core genes: CASP3, CASP4, GSDMB, and GZMA. Patients in the high-risk group showed a significantly shortened overall survival. The tumor microenvironment of the high-risk group exhibited immunosuppressive characteristics, including increased infiltration of immunosuppressive cells such as regulatory T cells, and upregulated expression of immune checkpoint molecules (e.g., ICOS, CTLA4, PDCD1) and immunosuppressive cytokines (e.g., TGFβ1, IL10). Single-cell transcriptomic analysis identified 18 cell clusters, and the expression of the 4 model genes in cancer cells was higher than that in adjacent non-tumor tissues, clarifying the distribution characteristics of these genes in different cell subtypes. This signature can effectively predict the prognosis of ccRCC patients, reflect the state of the tumor immune microenvironment, and provide candidate insights for immune checkpoint inhibitor therapy stratification in ccRCC.

### 4.2 PRGS in non-neoplastic diseases

For osteoarthritis (OA), a common degenerative joint disease, Chen et al. (69) integrated scRNA-seq and bulk RNA-seq data to construct a PRGS, aiming to unravel the regulatory role of pyroptosis in the pathological progression and prognosis of OA. This signature comprised two categories of core genes: one being 10 pyroptosis-related differentially expressed genes (PRDEGs), including CASP1, CASP3, CASP6, CASP8, GSDMB, NLRP1, NOD1, PLCG1, PRKACA, and PYCARD; and the other being 15 core hub genes, such as WAS, AGT, CTSD, APOE, CYBA, NCF1, ITGAM, AGTR1, CD74, CORO1A, ITGB2, PTPN6, THBS1, CD4, and CD14. Among them, PRDEGs, as core molecules of the pyroptosis pathway, directly reflected the activation state of pyroptosis-related transcriptional signatures in OA, and the pyroptosis score constructed based on these genes enabled effective prognostic stratification of patients. The 15 core hub genes, on the other hand, regulated reactive oxygen species metabolism, immune infiltration, and chondrocyte metabolism, and formed functional crosstalk with PRDEGs at the pathway level (e.g., phagosome and lysosome pathways), in the immune microenvironment, and across chondrocyte subtypes, jointly mediating the regulatory effect of pyroptosis on OA prognosis. This PRGS has uncovered the complex interaction mechanism between pyroptosis and the immune system in OA, providing a reliable molecular tool for OA prognostic assessment. Given the high clinical difficulty in distinguishing OA (a degenerative disease) from rheumatoid arthritis (RA, an autoimmune disease) and the heterogeneity in their molecular mechanisms, Zheng et al. (70) integrated scRNA-seq and RNA microarray data to construct a PRGS applicable to both diseases, addressing this critical clinical challenge. This signature also consisted of two categories of core genes. One category included 6 RNA modification-related disease-specific biomarkers, where OGN, TNFRSF11B, SCARA3, and PTN were OA-related biomarkers, and ADAMDEC1 and IGHM were RA-related biomarkers; these genes not only enabled highly specific differentiation between OA and RA but also effectively assessed the degree of disease progression and prognosis. The other category comprised 6 pyroptosis-RNA modification-associated hub genes (CXCL10, CXCL9, CCR7, CCL5, CXCL1, CCR2), whose core function was to dissect the synergistic regulatory mechanism among the pyroptosis pathway, RNA modification, and immune infiltration. By jointly participating in the regulation of inflammatory responses and the pyroptosis pathway, these two categories of PRGS directly influenced the trajectory of patients’ disease progression and prognostic outcomes. On one hand, this gene signature provides highly specific diagnostic biomarkers, resolving the key clinical need for differential diagnosis between OA and RA; on the other hand, it reveals the crosstalk mechanism among pyroptosis, RNA modification, and immune infiltration in the pathological process of arthritis, pointing out the direction for prognostic assessment of both diseases. Meanwhile, it also offers core resources for in-depth understanding of the molecular heterogeneity between degenerative OA and autoimmune RA.

Necrotizing enterocolitis (NEC) is an acute intestinal dysfunction disease characterized by inflammatory responses and cell death (including pyroptosis), and the degree of intestinal damage directly determines patient prognosis. To explore the role of pyroptosis in NEC and identify prognostic targets, Zhang et al. (71) integrated scRNA-seq and bulk RNA-seq data to construct a PRGS containing TREM1, TLN1, NOTCH2, MPZL1, and ADA. Based on scRNA-seq, they also discovered a novel macrophage subset, TREM1^+^CD163^+^, which was specifically enriched in NEC. This macrophage subset primarily communicated with other cells through the NAMPT/ITGA5/ITGB1 and CCL3/CCR1 pathways. Among the genes in the signature, the core gene TREM1 was highly expressed in the intestinal tissues of NEC patients; it exacerbates intestinal inflammation and tissue damage by activating the pyroptosis pathway, thereby promoting the pathological process and poor prognosis of NEC. This PRGS not only clarifies the critical role of TREM1^+^CD163^+^ macrophages in regulating pyroptosis in NEC but also reveals the potential value of TREM1 as a target for assessing disease progression and improving patient prognosis in NEC.

In the field of metabolic diseases, the core pathological features of type 2 diabetes (T2D) are insulin resistance and pancreatic islet dysfunction, and pyroptosis has been confirmed to be closely associated with islet cell damage. To analyze the association between pyroptosis and the pathological progression of T2D and establish effective prognostic stratification tools, Song et al. (72) integrated scRNA-seq and bulk RNA-seq to construct a T2D-related PRGS. The identified core genes were functionally divided into two interconnected modules supporting T2D prognostic evaluation. The first module consisting of GBP2, NLRP1, and NOD2 enabled accurate prediction of T2D onset risk (AUC = 0.968), which assisted early identification of populations at high risk of subsequent disease progression. The second module included 17 pyroptosis-related hub genes (INS, CHGA, GCG, GAD2, NEUROD1, PCSK1, ABCC8, GRIA2, CHGB, STMN2, GNAS, IAPP, CPE, KCNQ1, NKX2-2, SCG5, and SLC17A6) that primarily reflected long-term pathological alterations and prognosis in T2D, including islet dysfunction and immune microenvironment disorder. Functional analyses demonstrated that these hub genes were closely correlated with the infiltration of 13 immune cell subtypes and significantly associated with the activity of disease progression-related pathways, such as myc target signaling and the G2M checkpoint. Differences in pyroptosis scores further distinguished immune activation status, disease severity, and prognosis between T2D subgroups. Collectively, this PRGS serves as a reliable prognostic biomarker system: it dynamically reflects pyroptosis-driven islet injury, immune dysregulation, and disease deterioration, and provides a robust molecular basis for long-term prognostic assessment, disease progression monitoring, and high-risk patient stratification in T2D.

In the field of neurodegenerative diseases, the pathological progression of Alzheimer’s disease (AD) is closely associated with neuroinflammation and cellular pyroptosis, and clarifying PRGS is of great significance for AD prognostic assessment. Ding et al. (73) established a PRGS (including GSDMD, CHMP4B, CHMP4A, CHMP2A, HMGB1, and CASP3) based on bulk RNA-seq. They found the gene combination provided high accuracy and specificity for the clinical diagnosis of patients with AD. Moreover, CHMP4B served as the hub gene, and its expression negatively correlated with AD severity, enabling prognostic stratification of patients. Further investigation using scRNA-seq revealed that the proportion of microglial subsets in the brain tissues of AD patients was significantly increased, and these microglial subsets were in an immune-activated state. In activated microglia, the expression of GSDMD was significantly upregulated, while the expression of CHMP4B was notably downregulated, showing a negative correlation between the two. Immunofluorescence co-localization results also indicated that CHMP4B and GSDMD were co-localized in microglia surrounding Aβ plaques. Further studies demonstrated that overexpression of CHMP4B could inhibit the activation of GSDMD, reduced the release of LDH and inflammatory factors (IL-1β, IL-18), and improved mitochondrial function, ultimately alleviating the neuroinflammatory response mediated by microglial pyroptosis. In summary, this study confirms that the PRGS centered on CHMP4B is closely associated with AD prognosis, verifies the role of CHMP4B as a protective factor in AD, and reveals its molecular mechanism of regulating microglial pyroptosis by modulating GSDMD.

Notably, it should be emphasized that PRGS are computational readouts derived from transcriptomic profiles. High scores reflect transcriptional activation of pyroptosis-associated gene sets, but do not directly equal bona fide functional pyroptotic cell death. Complete pyroptotic cell lysis may result in cell loss during single-cell dissociation, which could further bias scoring results. An evidence hierarchy should be kept in mind when interpreting these prognostic models including transcriptional association and pathway enrichment, spatial co-localization, protein-level validation (e.g. western blot for cleaved GSDMD), *in vitro*/*in-vivo* functional perturbation assays, or independent cohort or prospective clinical validation. In addition, most existing PRGS models face common risks including model overfitting, confounding effects derived from immune-cell composition, and cohort-specific bias. The majority of these models are constructed from retrospective public datasets and lack rigorous external or prospective clinical validation. Therefore, they should not be regarded as clinically-ready tools for real-world patient management at present.

## 5 Single-cell sequencing provides insights in targeted pyroptosis therapeutic strategies

Previous therapeutic studies targeting pyroptosis have achieved significant progress: they have not only identified a series of potential therapeutic targets, including core molecules of the pyroptosis pathway and various regulatory molecules, but also confirmed that targeted intervention in the pyroptosis process can play a role in a variety of diseases, opening up a new direction for the treatment of related diseases. However, these studies still face the challenge of insufficient precision and individual adaptation during their translation to clinical practice. By analyzing the single-cell molecular characteristics related to pyroptosis, scRNA-seq focuses the previous broad exploration of mechanisms on the precise dimension of cell subtype specificity, providing a precise biological basis for the optimization of disease treatment strategies (**Table 4**).

**Table 4:** ScRNA-seq provides insights in targeted pyroptosis therapeutic strategies.

Therapeutic intervention category	Disease/model	Species/model & sample size	Data type & dataset	Pyroptosis-inference method/gene set	Orthogonal/functional validation	Independent-cohort validation	Evidence level	Key scRNA-seq findings	Intervention approaches & therapeutic significance	Ref
Targeting Core Pyroptosis Molecules										
NLRP3	RILI	Mouse: RILI model; mouse alveolar macrophage cell lines ANA-1, MH-S; scRNA-seq n=3	in-house scRNA-seq, public dataset GSE211713	Pyroptosis-related gene signatures curated from published literatures; FLICA-Annexin-V flow-cytometry pyroptotic cell phenotyping	flow cytometry, WB, qRT-PCR, Co-IP, mass-spectrometry, Duolink PLA; *in vitro* and *in vivo* MCC950 pharmacological intervention	No	T+P+F+A	AM are the first cell type to undergo pyroptosis, and pyroptosis levels correlate closely with the severity of acute radiation pneumonitis.	NLRP3 inhibitor MCC950 alleviates lung inflammation and fibrosis, providing a precise target for RILI.	(74)
GSDMD	AAA	Human: AAA patients n=84, healthy control n=95; Mouse: Ang-II/elastase-induced AAA model; HASMC cell lines; public mouse scRNA-seq dataset	in-house bulk RNA-seq, in-house untargeted metabolomics; public mouse scRNA-seq dataset	GSDMD as core pyroptosis effector gene; protein WB for N-GSDMD pyroptosis marker	WB, immunofluorescence, untargeted metabolomics, ChIP, dual-luciferase reporter assay; *in vitro* si-GSDMD/si-CHOP/si-CEBPB genetic perturbation in HASMC; VSMC-specific GSDMD-knockout mice *in vivo* AAA model; DFMO pharmacological intervention	No	T+P+F+A	GSDMD is significantly upregulated in VSMCs.	Specific GSDMD knockout or ODC1 inhibitor DFMO attenuates AAA formation.	(75)
	BBB damage	Mouse: LPS/Klebsiella-induced sepsis BBB-disruption model; primary mouse bEC, hBMEC cell lines; in-house and public scRNA-seq (exact count not reported)	in-house scRNA-seq (GSE211099); public scRNA-seq (GSE98816, GSE112436)	GSDMD/Caspase-11 as core pyroptosis effector genes; WB detection of N-GSDMD; SYTOX-green/LDH-release cell-death phenotyping	WB, immunofluorescence, electron microscopy, BBB permeability tracer assays; *in vitro* LPS-transfection-induced pyroptosis in primary bEC/hBMEC; multiple whole-body/cell-specific Gsdmd-KO, Casp11-KO mouse *in vivo* model; AAV-mediated endothelial-specific GSDMD rescue/nanobody inhibition assay	No	T+P+F+A	The bECs are highly sensitive to LPS, and GSDMD is a key mediator of BBB disruption.	bEC-specific GSDMD knockout or GSDMD-neutralizing nanobody (VHHGSDMD-1) can protect the BBB.	(76)
GSDME	Psoriasis	Human: public psoriasis skin specimens (exact count not reported); Mouse: IMQ-induced psoriasis-like dermatitis model; HaCaT human keratinocyte cell line	bulk RNA-seq: GSE13355, GSE14905, GSE30999, GSE85034, GSE53552, GTEx database, in-house mouse epidermal bulk RNA-seq; scRNA-seq: GSE162183	GSDME as core pyroptosis effector gene; WB detection of cleaved GSDME-NT fragment	WB, qRT-PCR, IHC, immunofluorescence, Co-IP, flow cytometry, ELISA, histopathological staining; *in vitro* sh-GSDME genetic perturbation in HaCaT keratinocytes; global Gsdme-KO and keratinocyte-specific Gsdme cKO *in vivo* mouse model	No	T+P+F+A	GSDME is highly expressed in keratinocytes and positively correlates with disease severity.	GSDME knockout alleviates skin inflammation, offering a novel target for psoriasis.	(77)
	Immunotherapy resistance in HCC	Human: pembrolizumab-treated HCC patient cohort (in-house scRNA-seq total n=26); Mouse: orthotopic/spontaneous ICB-resistant HCC mouse models (in-house single-cell multi-omics total n=12); Hepa1-6-PD-L1R, RIL-175-PD-1R HCC tumour cell lines	bulk RNA-seq: TCGA-LIHC, GSE148379; scRNA-seq, scATAC-seq, nanoscale ChIP-seq: in-house single-cell multi-omics datasets	GSDME as core pyroptosis effector gene; WB detection of cleaved GSDME-N fragment; LDH-release, HMGB1 secretion, 7-AAD/Annexin-V flow-cytometry for pyroptosis cellular phenotyping	WB, RT-qPCR, ChIP-seq, ChIP-qPCR, flow cytometry, ELISA; *in vitro* CRISPR-mediated STAT1-KO/GSDME-KO genetic perturbation in HCC tumour cells; multiple orthotopic and spontaneous ICB-resistant HCC *in vivo* mouse models; CXD101-ICB pharmacological combination intervention; CD8^+^T-cell/tumour cell co-culture cytotoxicity assay	No	T+P+F+A	High HDAC expression causes IFNγ signaling deficiency, leading to ICB resistance.	HDAC inhibitor CXD101 combined with ICB activates GSDME-mediated pyroptosis and reverses resistance.	(78)
Modulating Endogenous Molecules										
MARCH2	Myocardial I/R injury	Human: ischemic cardiomyopathy patient specimens total n=12; Mouse: myocardial I/R injury model (in-house scRNA-seq total n=12); NMCM, HL-1 cardiomyocyte cell lines	in-house mouse heart scRNA-seq dataset (OEP004577)	GSDMD as core pyroptosis effector gene; WB detection of cleaved GSDMD-N fragment; PI staining for cardiomyocyte death phenotyping	WB, qRT-PCR, co-IP, GST pull-down, ubiquitination assay, ELISA, immunofluorescence, live-cell imaging; *in vitro* si-MARCH2/si-MAVS/CRISPR-PGAM5-KO genetic perturbation in cardiomyocytes; MARCH2-KO mouse, AAV-mediated cardiac-specific MARCH2/PGAM5/MAVS overexpression or knockdown *in vivo* I/R model; hypoxia/reoxygenation (H/R) cell injury model	No	T+P+F+A	MARCH2 is elevated in ischemic myocardium, and its deficiency exacerbates NLRP3 inflammasome activation.	AAV9-mediated cardiac-specific MARCH2 overexpression inhibits pyroptosis and protects myocardium.	(79)
AhR	IBD	Human: ulcerative colitis patient specimens (public dataset total n=107); Mouse: DSS-induced colitis model (in-house scRNA-seq total n=6); Bone-marrow-derived macrophage (BMDM), RAW264.7 cell lines	bulk RNA-seq: GSE75214; scRNA-seq: in-house mouse colon scRNA-seq dataset (GSE242968)	GSDMD as core pyroptosis effector gene; WB detection of cleaved GSDMD-N fragment; PI staining, LDH-release assay for macrophage pyroptosis phenotyping	WB, qRT-PCR, ChIP, dual-luciferase reporter assay, ELISA, immunofluorescence, ASC-speck imaging; *in vitro* si-ODC1/ODC1 overexpression genetic perturbation in macrophages; global AhR-KO, myeloid-specific AhR conditional-KO *in vivo* DSS colitis model; I3C/spermine pharmacological intervention	No	T+P+F+A	AhR deficiency enhances macrophage pyroptosis and intestinal inflammation.	AhR ligand I3C or spermine supplementation inhibits NLRP3 assembly and relieves colitis.	(80)
LCN2	UC	Human: UC and control colonic mucosal specimens total n=39; Mouse: DSS-induced colitis model; NCM460 human intestinal epithelial cell line	bulk RNA-seq: GSE75214, GSE107499, GSE66407, GSE160804, GSE178753, GSE87466; scRNA-seq: GSE231993	GSDMD as core pyroptosis effector gene; WB detection of cleaved GSDMD-N fragment; PI staining, LDH-release assay for intestinal epithelial cell pyroptosis phenotyping	WB, qRT-PCR, IHC, immunofluorescence, 16S rRNA sequencing; *in vitro* si-LCN2 genetic perturbation in NCM460 cells; *in vivo* plasmid-mediated LCN2 overexpression/si-LCN2 silencing; GSDMD-KO DSS-colitis mouse model; IKK16 pharmacological inhibition assay	No	T+P+F+A	LCN2 is highly expressed in IECs in active UC and drives pyroptosis.	LCN2 silencing suppresses IECs pyroptosis and reduces intestinal damage.	(81)
Utilizing Exogenous Compounds										
MCDs	IVDD	Human: degenerated and non-degenerated intervertebral disc specimens (exact count not reported); Rat: needle-puncture-induced IVDD model; Human nucleus pulposus (NP) primary cells	bulk RNA-seq: in-house human NP-cell bulk RNA-seq; scRNA-seq: public dataset (GSE199866)	GSDMD as core pyroptosis effector gene; WB detection of cleaved GSDMD-N fragment; LDH release, NLRP3-ASC speck staining for NP-cell pyroptosis phenotyping	WB, qRT-PCR, immunofluorescence, SEM, seahorse mitochondrial respiration assay; *in vitro* H_2_O_2_-induced oxidative damage NP-cell model; needle-puncture IVDD rat *in vivo* model; MCDs catalytic nanodots/N-acetylcysteine pharmacological intervention	No	T+P+F+A	Fibrotic NP cells show the highest pyroptosis score, with ROS as a major trigger.	MCDs scavenge ROS, inhibit NP cell pyroptosis, and slow degeneration.	(82)
Kae	RA	Human: rheumatoid arthritis synovial specimens (exact count not reported); Mouse: collagen-induced arthritis (CIA) mouse model (DBA/1 mice)	bulk RNA-seq: GSE55235, GSE89408; scRNA-seq: GSE200815	GSDMD as core pyroptosis effector gene; immunofluorescence detection of cleaved GSDMD-N fragment; NLRP3/CASP1/GSDMD axis as key pyroptosis-related signature	WB, qRT-PCR, immunofluorescence, molecular docking, ELISA, flow cytometry, micro-CT, histopathological staining (HE, Safranin O-Fast green, TRAP); *in vivo* CIA mouse model; kaempferol pharmacological intervention	No	T+P+A	Pyroptosis pathways are aberrantly activated in synovial cells and linked to immune cell infiltration.	Kae directly binds to pyroptotic key proteins, inhibits pyroptosis, and restores immune balance.	(83)
JAK1/2 inhibitors	APOL1-associated kidney disease	Human: human kidney biopsy specimens; Human iPSC-derived kidney organoids, primary human adult kidney endothelial cells (AKECs, 3 donors), pooled HUVEC cell lines	bulk RNA-seq: GSE219185; scRNA-seq/snRNA-seq: GSE230848, GSE135663, GSE213030	GSDMD as core pyroptosis effector gene; WB detection of cleaved GSDMD-N fragment; pyroptosis-associated gene signature profiling	WB, ELISA, immunofluorescence, live-cell confocal imaging, Seahorse mitochondrial stress assay, digital spatial profiling; *in vitro* IFN-γ stimulation, JAK-STAT/pan-caspase pharmacological inhibition in organoids and primary endothelial cells; human kidney organoid disease model	No	T+P+F+O	IFN-γ prominently induces pyroptosis in renal endothelial cells.	JAK1/2 inhibitors (baricitinib) block the IFN-γ signaling axis and ameliorate renal injury.	(84)
Indirect Intervention										
Cell functional states modulation	Inflammation-related vascular diseases	Human: human atherosclerotic arterial specimens; Mouse: carotid artery ligation-induced neointimal hyperplasia mouse model; Primary mouse vascular smooth muscle cells (VSMCs), HEK293A cell lines	bulk RNA-seq: in-house mouse carotid artery bulk RNA-seq; scRNA-seq: in-house mouse carotid artery scRNA-seq (GSE194173)	GSDMD as core pyroptosis effector gene; WB detection of cleaved GSDMD-N fragment; pyroptosis-related gene signature profiling from scRNA-seq	WB, qRT-PCR, immunofluorescence, H&E staining, Co-IP, GST pull-down, PLA, CUT&RUN, dual-luciferase reporter assay, phagocytosis assay; *in vitro* si-Sox10/overexpression/mutant plasmid genetic perturbation in VSMCs; RGS5-KO, Myh11-CreERT2-mTmG reporter mouse, local perivascular si-RNA interference *in vivo* carotid ligation model	No	T+P+F+A	VSMCs transdifferentiate into macrophage-like subsets with enhanced pyroptosis.	Sox10 silencing suppresses transdifferentiation and pyroptosis, alleviating vascular inflammation.	(85)
	AML	Human: peripheral blood from healthy donors and AML patients; Mouse: NSG/NSG-SGM3 xenograft tumor models; Human expanded ILC2s, AML cell lines (MOLM13, U937, THP1), solid tumor cell lines	bulk RNA-seq: in-house bulk RNA-seq (GSE247206, GSE247207); scRNA-seq: in-house scRNA-seq (GSE247205)	GSDME as core pyroptosis effector gene; WB detection of cleaved GSDME-N fragment; combined pyroptosis-apoptosis cell-death phenotyping by time-lapse microscopy, Annexin-V/DAPI flow cytometry	WB, qRT-PCR, ELISA, flow cytometry, time-lapse live-cell imaging; *in vitro* CRISPR-GZMB-KO/GSDME-KO genetic perturbation in ILC2s and AML tumor cells; human ILC2-tumor cell co-culture cytotoxicity assay; multiple NSG xenograft *in vivo* tumor models with adoptive ILC2 transfer; TCGA-AML retrospective survival correlation analysis	No	T+P+F+A	The tumor microenvironment impairs ILC2 cytotoxicity and pyroptosis-inducing capacity.	Adoptive transfusion of Ex ILC2s, combined with MEK inhibitor trametinib, restores anti-tumor pyroptotic effects.	(86)
Gut Microbiota Regulation	Aging-related colitis	Human: IBD and healthy control fecal specimens (total n=88); Mouse: DSS-induced colitis, IL-10-KO, macrophage-specific GSDMD-KO mice; BMDMs, RAW264.7 macrophage cell lines	16S rDNA sequencing, untargeted metabolomics, scRNA-seq, bulk RNA-seq: in-house datasets (CRA019312, CRA019269, CRA019267; OMIX007519)	GSDMD as core pyroptosis effector gene; WB detection of cleaved GSDMD-N fragment; PI staining, LDH-release assay for macrophage pyroptosis phenotyping	WB, qRT-PCR, Co-IP, immunofluorescence, histopathological staining(H&E, PAS); *in vitro* LPS/ATP-induced macrophage pyroptosis model, NR4A1 agonist pharmacological intervention; fecal microbiota transplantation (FMT); macrophage-specific GSDMD-KO *in vivo* DSS-colitis mouse model; NDGA pharmacological intervention	No	T+P+F+A	Aging-associated dysbiosis reduces NDGA levels and exacerbates macrophage pyroptosis.	NDGA supplementation, fecal microbiota transplantation, or lactobacilli administration inhibits macrophage pyroptosis.	(87)
	Colitis	Mouse: DSS-induced colitis and *Citrobacter rodentium* infection mouse models; Primary intestinal intraepithelial lymphocytes (IELs)	16S rDNA sequencing, untargeted metabolomics, scRNA-seq: in-house mouse datasets	GSDMD as core pyroptosis effector gene; WB detection of cleaved GSDMD-N fragment	WB, qRT-PCR, flow cytometry, histopathological staining(H&E); *in vivo* neutrophil-depletion antibody treatment, co-housing, antibiotic-mediated microbiota depletion; DMF pharmacological intervention in DSS-colitis mouse model; *Citrobacter rodentium* bacterial infection model	No	T+P+F+A	Dysbiosis leads to a decrease in DMF, triggering GSDMD-mediated pyroptosis in intestinal IELs.	DMF supplementation or restoration of microbiota balance suppresses IELs pyroptosis.	(88)

Ref, references; RILI, radiation-induced lung injury; scRNA-seq, single-cell RNA sequencing; AMs, alveolar macrophages; AAA, abdominal aortic aneurysm; VSMCs, vascular smooth muscle cells; BBB, blood-brain barrier; bECs, brain endothelial cells; HCC, hepatocellular carcinoma; ICB, immune checkpoint blockade; I/R, ischemia/reperfusion; IBD, Inflammatory bowel disease; AhR, aryl hydrocarbon receptor; UC, ulcerative colitis; LCN2, Lipocalin-2; IECs, intestinal epithelial cells; MCDs, Mn-containing nanodots; IVDD, intervertebral disc degeneration; ROS, reactive oxygen species; NP, nucleus pulposus; Kae, kaempferol; RA, rheumatoid arthritis; IFN-γ, γ-interferon; AML, acute myeloid leukemia; ILC2s, group 2 innate lymphoid cells; Ex ILC2s, expanded ILC2s; NDGA, nordihydroguaiaretic acid; IELs, intraepithelial lymphocytes; DMF, Dimethyl fumarate.

T, transcriptional association; P, protein and/or mRNA level validation; F, *in vitro* functional perturbation; A, animal-model *in vivo* evidence; O, patient-derived tissue/organoid validation; R, retrospective clinical association; Pr, prospective clinical evidence. Combined codes (e.g., T+P) are used for multiple-layer evidence.

### 5.1 Therapeutic strategies targeting core molecules of the pyroptosis pathway

Core molecules of the pyroptosis pathway (e.g., NLRP3 inflammasome, Gasdermin family members GSDMD and GSDME) serve as key nodes regulating the pyroptotic process. Inhibition or specific knockout of these molecules has demonstrated clear therapeutic potential in various disease models.

As a critical initiating molecule of the pyroptosis pathway, abnormal activation of the NLRP3 inflammasome is a core link mediating pyroptosis and triggering inflammatory damage. In radiation-induced lung injury (RILI), Zhang et al. (74) utilized scRNA-seq to dissect the injury mechanism at the single-cell level. They found that alveolar macrophages (AMs) were the first cell type to undergo pyroptosis after radiation exposure, and AM pyroptosis mediated by the NLRP3/Caspase-1/GSDMD axis was the core mechanism of RILI. The level of AM pyroptosis was closely correlated with the Szapiel’s score for acute radiation pneumonitis. Treatment with MCC950, a specific NLRP3 inhibitor, significantly reduced the expression of GSDMD-N and cleaved-caspase-1, as well as pyroptosome formation in AMs by blocking the interaction between NLRP3 and ASC. This further decreased the infiltration of neutrophils and inflammatory monocytes, and the release of pro-inflammatory cytokines such as IL-1β and IL-6. In *in vivo* experiments, MCC950 not only alleviated acute radiation pneumonitis but also long-term inhibited radiation-induced pulmonary fibrosis (by reducing collagen deposition, suppressing fibroblast activation, and inhibiting interstitial macrophage infiltration). These findings suggest that targeting NLRP3-mediated AM pyroptosis may be an effective strategy for RILI treatment.

GSDMD is a core executor of the classical pyroptosis pathway, its activation causes membrane pore formation and triggers pyroptosis. Therapeutic strategies targeting GSDMD have shown application prospects in multiple disease fields. In AAA, Gao et al. (75) identified via single-cell transcriptomic analysis that GSDMD expression was upregulated in aortic vascular smooth muscle cells (VSMCs) of mice with angiotensin II (Ang II)-induced AAA. Specific knockout of GSDMD in VSMCs alleviated AAA formation by inhibiting the endoplasmic reticulum stress-CHOP signaling pathway, reducing ornithine decarboxylase 1 (ODC1) expression and putrescine synthesis, maintaining the contractile phenotype of VSMCs, and lowering inflammatory responses. However, supplementary putrescine reversed this protective effect, while treatment with the ODC1 inhibitor difluoromethylornithine (DFMO) also mitigated AAA. Additionally, population studies confirmed a significant correlation between plasma putrescine levels and AAA risk, indicating that regulating the GSDMD-putrescine axis to interfere with pyroptosis-related pathways is a potential direction for AAA treatment. GSDMD also plays a core role in blood-brain barrier (BBB) disruption, a key pathological process in central nervous system diseases. Wei et al. (76) performed single-cell sequencing on the brains of LPS-stimulated mice and found that brain endothelial cells (bECs) exhibited the most prominent response to circulating LPS. Moreover, activation of the caspase-11/GSDMD non-canonical inflammasome in bECs was the core mechanism underlying LPS-induced BBB disruption. To achieve precise intervention, the research team employed two specific targeting strategies: 1) generating bEC-specific GSDMD knockout mice by crossing bEC-specific Cre driver mice with GSDMD conditional knockout mice; 2) achieving bEC-specific GSDMD knockout in adult mice via AAV-BI30 virus (targeting bECs) -mediated delivery of Cre recombinase. Both strategies effectively blocked BBB disruption induced by LPS or Klebsiella pneumoniae infection without affecting the normal physiological function of GSDMD in other cells. Furthermore, the team developed a GSDMD-neutralizing nanobody (VHHGSDMD-1). Targeted delivery of this nanobody to bECs via the AAV-BI30 virus enabled it to specifically bind to the GSDMD-N fragment and block its membrane pore-forming activity, which also effectively inhibited BBB disruption caused by LPS or bacterial infection. This provides a candidate pre-clinical therapeutic strategy for central nervous system diseases associated with BBB damage.

As another important member of the Gasdermin family, GSDME not only functions via the classical pyroptosis pathway but also regulates inflammation in a pyroptosis-independent manner. Long et al. (77) analyzed psoriasis-related scRNA-seq datasets and found that GSDME was specifically overexpressed in keratinocytes of lesional skin from psoriasis patients. Its expression was positively correlated with disease severity (PASI score) and the core pro-inflammatory cytokine IL-17A. Mechanistically, GSDME upregulated inflammatory mediators such as IL-1β and CCL20 not through the classical pyroptosis pathway, but by binding to the transcription factors p65 and c-jun in its full-length form to promote their nuclear translocation. Systemic or keratinocyte-specific knockout of GSDME significantly alleviated imiquimod (IMQ)-induced psoriasiform dermatitis, improved abnormal proliferation and differentiation disorders of keratinocytes, and reduced neutrophil infiltration and inflammatory cytokine secretion. *In vitro*, GSDME knockdown in keratinocytes also inhibited p65/c-jun nuclear translocation and the expression of downstream inflammatory mediators. These results suggest that targeting GSDME, a pyroptosis-related molecule, may effectively alleviate psoriasis inflammation, providing a new target and strategy for psoriasis treatment. In HCC immunotherapy, GSDME modulation offers a novel breakthrough for overcoming ICB resistance. Tu et al. (78) combined scRNA-seq and single-cell assay for transposase-accessible chromatin sequencing (scATAC-seq) and found that HCC cells with high HDAC1/2/3 expression exhibited defective IFNγ signaling and poor responsiveness to ICB therapy. Based on this finding, they tested a combination therapy of the selective class I HDAC inhibitor CXD101 with ICB. Results showed that this combination activated the STAT1 pathway through epigenetic remodeling, induced GSDME expression, recruited IFNγ^+^GZMB^+^ cytotoxic lymphocytes, and promoted GSDME cleavage via a CD8^+^ T cell-mediated, STAT1-dependent mechanism, thereby triggering tumor cell pyroptosis. Notably, GSDME knockout completely abrogated the anti-tumor effects of CXD101-ICB combination therapy (including inhibiting tumor growth and prolonging survival) and blocked pyroptotic responses and IFNγ signaling. This suggests that targeting GSDME-mediated pyroptosis may constitute a key mechanism for reversing ICB resistance in HCC, providing mechanistic support for the clinical application of CXD101 combined with ICB.

### 5.2 Endogenous molecules and exogenous compounds: potential carriers for regulating pyroptosis signaling axes

Beyond directly targeting core molecules of the pyroptosis pathway, endogenous molecules (e.g., MARCH2, aryl hydrocarbon receptor, lipocalin-2) and exogenous compounds (e.g., carbonized Mn-containing nanodots, kaempferol, JAK inhibitors) can also exert therapeutic effects by regulating pyroptosis-related signaling axes, offering more diverse carrier options for pyroptosis intervention.

#### 5.2.1 Endogenous molecules as endogenous targets for disease treatment

Endogenous molecules can precisely regulate pyroptosis by directly acting on key proteins of the pyroptosis pathway or modulating metabolite synthesis, serving as endogenous targets for disease treatment.

For myocardial ischemia-reperfusion (I/R) injury, Liu et al. (79) found via scRNA-seq analysis of myocardial tissue from I/R-injured mice that the E3 ubiquitin ligase MARCH2 was upregulated in ischemic cardiomyocytes. Moreover, MARCH2 deficiency exacerbated NLRP3 inflammasome activation in cardiomyocytes. Mechanistically, MARCH2 directly interacted with PGAM5 and promoted its degradation via K48-linked polyubiquitination, reducing the formation of liquid-liquid phase separation condensates between PGAM5 and MAVS. This consequently inhibited NLRP3 inflammasome activation and cardiomyocyte pyroptosis. Myocardial-specific overexpression of MARCH2 mediated by AAV9 significantly alleviated I/R-induced pyroptosis and myocardial damage. These results confirm that regulating the MARCH2/PGAM5/MAVS/NLRP3 axis to interfere with pyroptosis is a key mechanism for protecting the myocardium against I/R injury, providing a new endogenous target for myocardial I/R injury treatment. In inflammatory bowel disease (IBD) research, the role of the aryl hydrocarbon receptor (AhR), an important environmental-sensing transcription factor, in pyroptosis regulation has been gradually elucidated. Gao et al. (80) performed single-cell sequencing on CD45^+^ immune cells in the intestinal lamina propria of colitis mice and found that AhR deficiency led to an increased proportion of monocyte-macrophage lineage cells, enrichment of pro-inflammatory subtypes, and upregulated expression of NLRP3 inflammasome-related genes and pyroptosis-associated cytokines. This confirms that AhR deficiency exacerbates macrophage pyroptosis. Mechanistically, AhR promoted the transcription of ornithine decarboxylase 1 (Odc1) by directly binding to xenobiotic response elements (XRE1, XRE2) in the Odc1 promoter, enhancing polyamine (putrescine, spermidine, spermine) biosynthesis. Among these polyamines, spermine inhibited NLRP3 inflammasome assembly by suppressing K^+^ efflux, thereby inhibiting macrophage pyroptosis. Oral supplementation with the AhR precursor ligand indole-3-methanol (I3C) or direct spermine supplementation significantly inhibited macrophage pyroptosis and alleviated intestinal inflammatory damage in dextran sulfate sodium (DSS)-induced colitis mice, providing a new target and strategy for IBD treatment. Lipocalin-2 (LCN2), a potential fecal marker for UC, provides a novel entry point for UC treatment through its association with pyroptosis. Yang et al. (81) analyzed colonic mucosal cells from UC patients using scRNA-seq and found that LCN2 was primarily expressed in intestinal epithelial cells (IECs). LCN2 expression was significantly higher in patients with active UC than in those in remission and healthy individuals, and its high expression was strongly associated with pyroptosis pathway enrichment. Mechanistically, LCN2 activated the NF-κB signaling pathway (promoting the phosphorylation of IKKα/β, IκBα, and p65, and nuclear translocation of p-p65), upregulated NLRP3 inflammasome expression, induced Caspase-1 activation and GSDMD cleavage, triggered IEC pyroptosis, and aggravated intestinal inflammation. *In vivo* experiments showed that after eliminating intestinal flora interference via antibiotic treatment, tail vein injection of LCN2 siRNA to silence LCN2 reduced the expression of pyroptosis markers in DSS-induced colitis mice and alleviated symptoms such as colon shortening and epithelial damage. This confirms that targeting LCN2 can inhibit IEC pyroptosis, offering a potential target for UC treatment.

#### 5.2.2 Exogenous compounds as tools for pyroptosis targeting therapy

A series of exogenous compounds exert therapeutic potential by interfering with pyroptosis signaling pathways, leveraging their unique physicochemical properties or molecular binding capabilities, thus providing abundant exogenous tools for pyroptosis-targeted therapy.

In intervertebral disc degeneration (IVDD) research, Sun et al. (82) found through scRNA-seq that fibrotic nucleus pulposus (NP) cells exhibited the highest pyroptosis score among all cell subsets, with significant enrichment of pyroptosis-related genes. Additionally, NLRP3 inflammasome expression increased with IVDD severity. Further studies confirmed that excessive accumulation of reactive oxygen species (ROS) was the core trigger for activating this pyroptosis pathway. Based on this, the authors developed carbonized Mn-containing nanodots (MCDs), which possess excellent superoxide dismutase (SOD)-, catalase (CAT)-, and peroxidase (POD)-like multi-enzymatic activities. Their antioxidant efficacy surpassed that of the classical antioxidant N-acetylcysteine (NAC), as they reduced H_2_O_2_-induced NP cell pyroptosis, protected mitochondrial function, and restored the intervertebral disc microenvironment homeostasis. In a puncture-induced rat IVDD model, MCDs alleviated IVDD in a dose-dependent manner (restoring disc height, improving MRI signals, and reducing ECM degradation) without obvious toxic side effects. These results confirm that the strategy of inhibiting pyroptosis via ROS scavenging by MCDs exhibits IVDD-alleviating potential in both *in vitro* and *in vivo* experiments, providing a new direction and candidate preclinical regimen for IVDD treatment. In rheumatoid arthritis (RA) research, the mechanism by which kaempferol (Kae), a natural flavonoid, regulates pyroptosis has been gradually clarified. He et al. (83) analyzed synovial tissue samples from RA patients using scRNA-seq and found that key genes of the pyroptosis-related NLRP3/Caspase-1/GSDMD axis were abnormally expressed in synovial cells (chondrocytes, macrophages, T cells) and closely correlated with immune cell infiltration. Molecular docking and functional experiments confirmed that Kae bound to NLRP3, Caspase-1, and GSDMD via hydrogen bonding and hydrophobic interactions. In a collagen-induced arthritis (CIA) mouse model, Kae inhibited pyroptosis in joint tissue cells, regulated T cell immune homeostasis (reducing the CD4^+^/CD8^+^ T cell ratio and CD4^+^ effector memory T cells, while increasing Tregs), and alleviated joint inflammation and bone destruction. These findings confirm that Kae inhibits pyroptosis and modulates T cell activation by regulating the NLRP3/Caspase-1/GSDMD axis, providing a new mechanism and potential strategy for RA treatment. In kidney disease research, JAK inhibitors have shown therapeutic potential by targeting and regulating the pyroptosis pathway. Juliar et al. (84) analyzed human kidney organoids and patient kidney samples using scRNA-seq and found that IFN-γ induced the expression of pyroptosis-related genes in multiple cell types of kidney organoids (podocytes, renal tubular cells, endothelial cells), with more prominent activation of the pyroptosis pathway in endothelial cells. Concurrently, IFN-γ upregulated APOL1 expression. Treatment with JAK1/2 inhibitors (e.g., baricitinib) blocked the JAK/STAT signaling pathway, inhibited IFN-γ-induced APOL1 expression and upregulation of pyroptosis-related genes, and rescued endothelial network degradation. In samples from patients with COVID-19-associated nephropathy, proteinuric nephropathy, and collapsing glomerulopathy, high expression of IFN signaling and pyroptosis-related genes was correlated with renal function deterioration. This suggests that targeting the IFN-γ/JAK/STAT-pyroptosis axis with JAK inhibitors may be a potential therapeutic strategy for kidney diseases, particularly APOL1-associated nephropathy.

### 5.3 Indirect pyroptosis regulation: innovative strategies based on cell function optimization and gut microbiota interaction

In addition to directly targeting the pyroptosis pathway or regulating pyroptosis signals via molecular carriers, indirect intervention in the pyroptotic process can be achieved by modulating cell functional states (e.g., transcription factor-mediated cell phenotypic transition, optimization of immune cell killing function) or leveraging interactions between gut microbiota (and their metabolites) and the host. These strategies provide more diverse and innovative ideas for treating related diseases.

Modulating transcription factor activity to remodel cell phenotypes is a key strategy for indirect pyroptosis regulation. In a mouse model of intimal hyperplasia, Xu et al. (85) identified via scRNA-seq that VSMCs could transdifferentiate into macrophage-like VSMCs with high expression of C3 and Cd74. Pyroptosis-related genes were significantly upregulated in this cell subset, and the transcription factor Sox10 was highly expressed, exhibiting a positive correlation with the accumulation of macrophage-like VSMCs and pyroptosis levels. Mechanistically, tumor necrosis factor α (TNF-α) induced phosphorylation of Sox10 at the S24 site via the PI3K/AKT signaling pathway, promoting its lactylation and activation, which in turn drove VSMC transdifferentiation. Silencing Sox10 or using a Sox10-S24A inactive mutant (incapable of phosphorylation-activated) reduced VSMC transdifferentiation into macrophage-like VSMCs, thereby inhibiting pyroptosis and ultimately alleviating vascular inflammation and intimal hyperplasia. These findings suggest that regulating Sox10-mediated VSMC transdifferentiation to indirectly modulate pyroptosis is a potential therapeutic direction for inflammation-related vascular diseases (e.g., atherosclerosis, vascular restenosis). Cell-based immunotherapy is revolutionizing the treatment paradigm for various cancers, and scRNA-seq provides a precise molecular basis for optimizing immune cell therapeutic strategies. Li et al. (86) performed single-cell transcriptomic analysis on peripheral blood group 2 innate lymphoid cells (ILC2s) from healthy donors (HD ILC2s), ILC2s from acute myeloid leukemia (AML) patients (AML ILC2s), *in vitro* expanded ILC2s (Ex ILC2s), and *in vivo* tracked mouse ILC2s (Traced ILC2s). They confirmed that Ex ILC2s retained a 97.36% pure ILC2 phenotype after *in vitro* expansion, ensuring the safety of cell therapy. Further analysis revealed significantly downregulated expression of DNAM-1 and granzyme B (GZMB) in AML patient-derived ILC2s, uncovering a mechanism by which tumors achieve immune escape via CD155-mediated DNAM-1 degradation. Concurrently, scRNA-seq verified that co-culture of Ex ILC2s with tumor cells upregulated GZMB expression and activated the AKT/FOXO1 signaling pathway, providing transcriptional evidence for the pyroptosis regulatory mechanism. Based on these findings, the team established an efficient *in vitro* expansion system for human ILC2s and confirmed that Ex ILC2s could bind to CD112/CD155 on tumor cells via DNAM-1, activating GZMB secretion. Specifically, Ex ILC2s induced pyroptosis by cleaving GSDME in GSDME^+^ tumor cells and triggered apoptosis by cleaving caspase 3 in GSDME^-^ tumor cells. *In vivo* experiments showed that adoptive transfer of Ex ILC2s significantly inhibited the growth of tumors such as AML, pancreatic cancer, and glioblastoma, prolonged the survival of tumor-bearing mice, and caused no toxicity such as graft-versus-host disease (GVHD). Furthermore, downregulating CD155 expression in tumor cells using the MEK inhibitor trametinib restored DNAM-1 expression and pyroptosis-inducing capacity of Ex ILC2s. This strategy provides a novel solution for overcoming tumor immune escape and optimizing adoptive immunotherapy efficacy.

Gut microbiota regulate key pyroptosis pathways via interactions between their metabolites and host cells; meanwhile, restoring gut microbiota homeostasis can indirectly inhibit excessive pyroptosis. This direction has become an important intervention approach for intestinal inflammatory diseases. Nordihydroguaiaretic acid (NDGA), a gut microbiota metabolite: Wang et al. (87) analyzed CD45^+^ immune cells in the intestinal lamina propria using scRNA-seq and found that age-related gut microbiota dysbiosis (characterized by reduced lactobacilli) led to decreased intestinal NDGA levels. This further caused significant enrichment of pro-inflammatory macrophages and neutrophils in colitis models, exacerbating macrophage pyroptosis. Mechanistically, NDGA inhibited macrophage pyroptosis by suppressing the GSDMD/NR4A1/NLRP3 signaling axis. Oral NDGA supplementation dose-dependently alleviated DSS- or IL-10 deficiency-induced colitis and improved intestinal barrier integrity. Importantly, fecal microbiota transplantation (transferring gut microbiota from young mice) or supplementation with specific lactobacilli (e.g., Lactobacillus johnsonii) restored intestinal NDGA levels and inhibited macrophage pyroptosis. This study provides an innovative “microbiota-metabolite-pyroptosis” regulatory direction for treating age-related IBD. Dimethyl fumarate (DMF), another gut microbiota metabolite: Chen et al. (88) analyzed intestinal intraepithelial lymphocytes (IELs) via scRNA-seq and found that CD177^+^ neutrophil deficiency led to significant enrichment of TCRγδ^+^CD8αα^+^ IELs in colitis model mice. These IELs highly expressed pyroptosis-related genes and pro-inflammatory cytokines, exhibiting a GSDMD-mediated pyroptotic phenotype. Concurrently, this was accompanied by gut microbiota dysbiosis (reduced fumarate-producing bacteria and increased fumarate-consuming bacteria) and decreased DMF levels. DMF deficiency exacerbated GSDMD-mediated pyroptosis of TCRγδ^+^CD8αα^+^ IELs, while oral DMF supplementation significantly inhibited this pyroptosis by suppressing GSDMD cleavage and reducing IL-1β/IL-18 release. It also improved gut microbiota dysbiosis and alleviated colitis symptoms induced by DSS or Citrobacter rodentium (CR) infection. Additionally, eliminating dysbiotic microbiota with antibiotics or restoring microbiota homeostasis via co-housing with wild-type mice also inhibited pyroptosis of TCRγδ^+^CD8αα^+^ IELs. This study further confirms the core role of gut microbiota and their metabolites in pyroptosis regulation, providing a new multi-faceted strategy for IBD treatment.

Collectively, these preclinical findings provide mechanistic insights into pyroptosis-oriented therapeutic strategies. Nevertheless, most pyroptosis-targeted interventions summarized in this section are validated using *in vitro* cellular assays and animal models, with only a small proportion supported by patient-derived organoid data. All these studies remain in the preclinical stage and lack validation from prospective clinical trials. On the other hand, key pyroptosis-related molecules such as GSDMD and NLRP3 exert context- and cell-type-dependent dual functions. They may promote anti-tumor immunity in certain scenarios, whereas they can also mediate excessive inflammatory tissue injury in other settings. Therapeutic blockade of pyroptosis carries potential risks, including compromised host antimicrobial defense and attenuated anti-tumor immune responses. Such double-edged-sword effects deserve careful consideration for future translational development.

## 6 Cross-disease synthesis and methodological appraisal

The preceding sections have systematically elaborated the applications of scRNA-seq in pyroptosis research organized by disease entity. To reinforce the systems immunology perspective of this review and move beyond a descriptive disease-by-disease catalogue, this section integrates conserved pyroptotic mechanisms across pathological contexts, addresses context-dependent and contradictory findings, and evaluates the methodological limitations and evidence hierarchy of the current research landscape.

Across diverse diseases, several core cellular and molecular features of pyroptosis consistently emerge from single-cell transcriptomic studies. At the cellular level, monocyte-macrophage lineage cells are the most widely implicated pyroptotic population, with consistently elevated pyroptotic activity across neoplastic, inflammatory, autoimmune and vascular disorders. Epithelial cells and endothelial cells also recurrently undergo pyroptosis as key structural and barrier cell types, while CD8^+^ T cells and NK cells serve as conserved effectors mediating anti-tumor immunity via pyroptosis-related mechanisms. At the molecular level, the canonical NLRP3/Caspase-1/GSDMD axis and its downstream effector cytokine IL-1β constitute the most universally activated pyroptotic signaling cascade. At the microenvironmental level, a stereotyped pathological cascade, pyroptotic cell death, release of pro-inflammatory mediators and DAMPs, myeloid cell recruitment, fibroblast activation, and excessive extracellular matrix deposition, drives fibrotic remodeling across multiple chronic inflammatory and neoplastic diseases. Prognostic pyroptosis-related gene signatures also share a common analytical framework: scRNA-seq is used to identify cell-type-specific core genes, which are then integrated with bulk RNA-seq cohorts to construct risk stratification models.

Despite these shared mechanistic themes, the functional outcomes of pyroptosis are highly context-dependent, with apparently contradictory findings across disease settings. Most notably, the prognostic association of pyroptosis diverges across cancer types: high pyroptotic activity correlates with favorable survival and improved immunotherapy response in metastatic melanoma, where it is driven by tumor-infiltrating immune cells, but predicts poor prognosis in glioblastoma, hepatocellular carcinoma and clear cell renal cell carcinoma, where it contributes to immunosuppressive microenvironment remodeling. Gasdermin family proteins also exhibit context-specific functions: GSDMD drives host antimicrobial defense in myeloid cells but mediates pathological tissue damage in structural cells such as vascular smooth muscle cells and endothelial cells; GSDME can even exert pro-inflammatory effects via transcriptional regulation independent of its classic pore-forming function, as observed in psoriasis. Furthermore, pyroptosis can shape either immunostimulatory or immunosuppressive microenvironments depending on duration and tissue context: acute pyroptosis enhances immune clearance of pathogens or malignant cells, while chronic pyroptosis in the tumor microenvironment promotes immune evasion.

Interpretation of these findings requires careful consideration of inherent disparities between human clinical studies and preclinical animal models. First, there are species-specific differences in pyroptotic molecular machinery: the non-canonical pyroptosis pathway is mediated by caspase-11 in mice but caspase-4/5 in humans, and surface markers defining macrophage and T cell subsets are not fully conserved across species. Second, there is a marked asymmetry in validation depth: nearly all functional perturbation studies, including gene knockout and pharmacological intervention, are performed in mouse models or cultured cell lines, while most human studies are limited to transcriptomic correlation or protein-level expression analysis without causal verification. Third, differences in tissue processing introduce systematic bias: mouse tissues are typically processed fresh with standardized protocols, whereas human clinical specimens often experience variable cold ischemia time and preservation-related artifacts; moreover, the genetic homogeneity of induced animal disease models cannot recapitulate the etiological and clinical heterogeneity of human diseases.

Notable technical and analytical limitations further affect the comparability and reliability of single-cell pyroptosis studies. In terms of experimental limitations, most scRNA-seq cohorts have relatively small sample sizes, increasing susceptibility to batch effects and individual selection bias. Dissociation-induced bias is another pervasive issue: the enzymatic and mechanical tissue dissociation process can upregulate cellular stress and inflammatory gene expression, producing false-positive pyroptotic signals; conversely, fully lysed pyroptotic cells are lost during dissociation and filtration, leading to systematic underestimation of pyroptotic activity in tissue samples. In terms of analytical heterogeneity, there is currently no community-wide standardized ontology for annotation of pyroptosis-relevant cell subsets, with variable clustering resolution and nomenclature across studies. Pyroptosis-related gene sets also vary widely in gene composition and curation source, and multiple divergent scoring algorithms are applied across studies, making direct cross-study comparison of pyroptosis scores challenging.

Finally, it is critical to distinguish conclusions based solely on transcriptomic inference from those supported by orthogonal functional validation. The majority of initial findings regarding cell-type-specific pyroptosis heterogeneity and intercellular communication crosstalk are derived from transcriptomic profiling and pathway enrichment analysis, representing associative rather than causal evidence. Approximately half of the reviewed studies include protein-level validation of key pyroptosis markers such as cleaved GSDMD or mature IL-1β. Around one-third include *in vitro* functional perturbation assays, such as gene knockout or pharmacological inhibition paired with pyroptosis functional readouts. Most mechanistic and therapeutic studies include validation in animal disease models, while prognostic gene signatures are typically validated in retrospective clinical cohorts. Overall, core pathways such as NLRP3/GSDMD-mediated macrophage pyroptosis have robust multi-level validation, but most novel cell subsets and context-specific mechanisms identified by scRNA-seq remain at the transcriptomic association level, and no pyroptosis-targeted therapeutic strategy has yet been supported by prospective clinical trials.

## 7 Conclusion and future prospects

This review mainly focuses on the application of scRNA-seq technology in pyroptosis research. From four core dimensions: analysis of pyroptosis heterogeneity, microenvironment remodeling, construction of prognosis-related gene signatures, and insights into targeted therapeutic strategies, it comprehensively summarizes the key progress achieved by this technology in advancing pyroptosis research from mechanism exploration toward preclinical therapeutic development (**Figure 1**). In terms of pyroptosis heterogeneity, scRNA-seq breaks through the limitations of traditional population-level detection and reveals the cell subtype-specific and state-dependent characteristics of pyroptosis in different diseases. Specifically, there are significant differences in pyroptosis-related transcriptional activity between immune cells (e.g., CD8^+^ T cells, macrophages) and non-immune cells (e.g., epithelial cells, fibroblasts), and the mechanisms of action of key molecules (e.g., GSDMD, GSDME, NLRP3) exhibit heterogeneity across diseases and cell types. In the field of microenvironment remodeling, this technology provides evidence that pyroptosis is associated with pro-inflammatory polarization or immunosuppression of the immune microenvironment, as well as abnormal ECM remodeling and angiogenesis in the stromal microenvironment, by releasing inflammatory factors and regulating intercellular communication. This provides an interactive perspective of the microenvironment for understanding disease progression. In terms of prognosis assessment, pyroptosis-related gene signatures constructed by integrating scRNA-seq and bulk RNA-seq represent candidate transcriptomic biomarkers for prognostic risk stratification. In terms of therapeutic strategies, based on the molecular mechanisms elucidated by scRNA-seq, strategies such as targeting core pyroptosis molecules (e.g., the NLRP3 inhibitor MCC950), regulating endogenous molecules (e.g., MARCH2), utilizing exogenous compounds (e.g., carbonized Mn-containing nanodots), and indirect intervention (e.g., gut microbiota modulation) have provided candidate preclinical therapeutic directions for various diseases.

Notably, pyroptosis is not an independent cell death process; instead, it forms an intricate interaction network with other cell death modalities such as autophagy, apoptosis, necroptosis, and ferroptosis (34, 89–94). These modalities are collectively enriched in the pathological progression of diseases and regulate cell fate and disease development directions through multi-dimensional crosstalk. For instance, autophagy-related genes can regulate pyroptosis, when autophagic function is impaired, it can activate pyroptosis-related inflammasomes through the accumulation of AIM2 protein, or affect the activity of pyroptotic pathways via the regulation of mitochondrial function (91, 92). Apoptosis and pyroptosis can be converted into each other under specific conditions: molecules represented by CASP3 not only participate in the execution of apoptosis but also switch the original apoptotic process to pyroptosis by cleaving members of the Gasdermin family (93). Conversely, inflammatory factors released after the upregulated expression of pyroptosis-related genes can also induce apoptosis in a reverse manner by activating the NF-κB signaling pathway (94). In autoimmune diseases, ferroptosis and pyroptosis exhibit interaction characteristics of “primary-secondary synergy” or “single dominance” (34). Furthermore, some novel cell death modalities (e.g., PANoptosis) directly integrate the key molecular features of pyroptosis, apoptosis, and necroptosis, which further reflects the in-depth association among various cell death modalities (95). This multi-dimensional interaction network not only determines the overall pattern of cell death in diseases (e.g., dominated by inflammatory death or non-inflammatory death) and the intensity of inflammatory responses but also directly correlates with critical pathological processes such as the remodeling of the tumor immune microenvironment and the development of therapeutic resistance. It thus provides important references for the disease mechanisms and the development of therapeutic targets.

Building upon the above-mentioned crosstalk among diverse programmed cell-death modalities, from a systems-immunology perspective, pyroptosis represents a multi-scale biological event that cannot be fully understood by merely cataloging disease-specific observations. Complex crosstalk exists across molecular signaling pathways, cell functional states, local tissue niches, organism-level phenotypes, and therapeutic responses. Pyroptotic signaling originating from distinct cell subsets propagates within tissue microenvironments, shapes local immune or stromal reactions, and further translates into systemic organismal phenotypes, which ultimately determine patient responses to anti-inflammatory or anti-tumor therapies. Multi-layer omics integration provides a feasible conceptual framework to untangle such complex immune-relevant behaviors. Combining scRNA-seq (cell-level transcript information), spatial transcriptomics (spatial tissue localization), single-cell proteomics (protein activation status), metabolomics (inflammatory metabolite landscape), together with clinical phenotypic datasets, enables us to connect molecular-level pyroptotic events to cell-state transition, tissue-level microenvironment remodeling, organismal phenotypes, and clinical treatment outcomes. Such multi-modal integration helps disentangle cause-effect relationships that cannot be resolved by single-layer transcriptomic profiling alone.

Nevertheless, multiple controversies and translational gaps remain in this field. As synthesized in Section 5, core constraints include context-dependent functional duality of pyroptosis, species discrepancies between preclinical models and clinical specimens, pervasive technical artifacts, unstandardized analytical workflows, and the predominance of transcriptomic correlation over causal functional validation. Community-wide unified reporting standards and orthogonal functional validation are needed to improve cross-study comparability. From a translational perspective, major bottlenecks also include high sequencing costs, lack of prospective clinical validation for pyroptosis-related gene signatures, and technical barriers to cell-subset-specific *in vivo* targeted delivery.

Testable standardized workflows are therefore required for follow-up research. Public benchmark datasets including TCGA, GEO and the Human Cell Atlas can be adopted for initial signature screening and cross-cohort comparison. A layered experimental validation strategy is recommended, covering *in vitro* cellular assays, patient-derived organoids and genetically modified animal models. Predefined validation endpoints include molecular readouts such as cleaved gasdermin detection, LDH release and mature IL-1β/IL-18 secretion at the cellular level, as well as clinical endpoints including long-term survival, disease progression and treatment response in prospective patient cohorts. Building on such standardized frameworks, future research should further dissect these interactive mechanisms by overcoming current technical constraints via integrative multi-omics innovation. Combining single-cell proteomics, single-cell metabolomics, and spatial transcriptomics enables the construction of a multidimensional pyroptosis regulatory network spanning transcription, protein activity, metabolism and spatial localization, to define the molecular machinery and spatiotemporal dynamics governing crosstalk between pyroptosis and other regulated cell death pathways. At the clinical translation level, pyroptosis-targeted therapeutic strategies informed by scRNA-seq-derived mechanistic findings can evolve toward precision and combinatorial regimens. Disease-specific multi-target combination therapies can be rationally designed, while pyroptosis-related gene signatures support precise patient stratification. Profiling crosstalk among cell death programs in lesion tissues also provides dynamic monitoring biomarkers to facilitate individualized regimen optimization.

Overall, scRNA-seq technology has opened a new “single-cell resolution” perspective for pyroptosis research, and its in-depth integration with multi-omics technologies will continuously unlock the underlying mechanisms of the interaction between pyroptosis and other programmed cell death modalities. In the future, with the continuous advancement of technological iteration and clinical translation, precision therapeutic strategies based on pyroptosis regulation are expected to be applied in more disease fields, providing a more efficient and targeted new paradigm for the diagnosis and treatment of inflammation-related diseases and tumors.
